# Supplementary material for: The Molecular Docking of MAX Fungal Effectors with Plant HMA Domain-Binding Proteins
Source: Int J Mol Sci. 2023 Oct 16;24(20):15239. doi: 10.3390/ijms242015239 (PMC10607590; doi:10.3390/ijms242015239)

The Molecular docking of MAX fungal effectors with plant HMA domain binding proteins

Lina Rozano [1,2], James K. Hane [2,3], and Ricardo L. Mancera [1,2]*

[1]Curtin Medical School, Curtin Health Innovation Research Institute, GPO Box U1987, Perth WA 6845, Australia

[2]Curtin Institute for Data Science, Curtin University, GPO Box U1987, Perth WA 6845, Australia

[3]Centre for Crop and Disease Management, School of Molecular and Life Sciences, Curtin University, GPO Box U1987, Perth WA 6845, Australia

*Correspondence: R.Mancera@curtin.edu.au (R. L. M.)

Supplementary Materials

Table S1: List of available experimental structures of effector proteins in complex with their respective host receptor binding partner (bound) used in benchmarking studies.

**Structural family**	**Fungal effector**	**Host receptor binding partner**	**PDB ID (chain from plant:chain from effector)**
MAX-like	Avr1CO39	RGA5	5ZNG A:C
	AvrPia	PikpHMA	6Q76 A:B
	AvrPikC	PikhHMA	7A8X B,E:C,F
	AvrPikD	PikmHMA	6FU9 A,C:B,D
	AvrPikE	PikmHMA<br>PikpHMA<br>PikpHMA^{NK-KE}	6FUB A:B<br>6G11 B,E:C,F<br>6R8M F:G
	AvrPikF	OsHIPP19	7B1I B:C
	ApikL2F	sHMA94	7NMM A,B,C,D,E,F,G,H:I,J,K,L,M,N,O,P

Table S2. Top 10 best DockQ scores, and rankings based on ZDOCK and ZRANK scores. Rankings shown in bold font indicate a top-10 pose for the respective scoring function.

Effector-host receptor binding partner complex	DockQ ranking	DockQ score	ZDOCK ranking	ZDOCK score	ZRANK ranking	ZRANK score
Avr1CO39-RGA5 (5zngAC)	1	0.967	756	34.19	35	-74.5563
	2	0.961	12	42.07	28	-75.754
	3	0.933	155	37.24	103	-68.3334
	4	0.933	107	37.97	207	-63.7273
	5	0.931	105	37.98	84	-69.7212
	6	0.928	1157	33.37	165	-65.6112
	7	0.918	380	35.56	43	-73.7114
	8	0.912	43	39.69	**1**	-89.5292
	9	0.895	128	37.55	72	-70.6197
	10	0.883	259	36.3	47	-73.1283
AvrPia-PikpHMA (6q76AB)	1	0.962	**3**	42.47	212	-64.0926
	2	0.942	209	36.35	157	-66.5047
	3	0.941	289	35.67	156	-66.5239
	4	0.926	241	36.06	162	-66.1953
	5	0.917	42	39.27	149	-66.8722
	6	0.899	33	39.67	137	-67.4449
	7	0.893	588	34.05	296	-60.2427
	8	0.886	854	33.25	99	-69.7505
	9	0.882	131	37.29	672	-49.3354
	10	0.875	909	33.12	152	-66.6196
AvrPikC-PikhHMA (7a8xBC)	1	0.917	**2**	60.16	**1**	-88.0183
	2	0.91	**1**	61.74	64	-70.0846
	3	0.888	11	53.75	13	-79.7873
	4	0.888	**9**	54.67	43	-72.5972
	5	0.886	**5**	57.62	350	-55.8018
	6	0.883	13	51.89	144	-64.4398
	7	0.878	36	46.23	116	-66.0273
	8	0.878	**6**	57.58	**4**	-83.0148
	9	0.874	27	47.89	284	-58.2618
	10	0.871	15	50.72	14	-79.3699
AvrPikC-PikhHMA (7a8xEF)	1	0.919	**1**	73.13	25	-85.0809
	2	0.916	**2**	69.6	22	-85.3532
	3	0.909	18	57.03	131	-68.1456
	4	0.904	**8**	60.38	**1**	-109.556
	5	0.888	**4**	62.24	11	-90.3229
	6	0.88	20	56.2	171	-65.9198
	7	0.872	**5**	62.03	43	-79.4627
	8	0.865	15	58.39	689	-47.1574

	9	0.859	13	58.81	138	-67.4861
	10	0.856	32	51.31	26	-84.6148
AvrPikD-PikmHMA (6fu9AB)	1	0.942	**9**	59.69	19	-92.2094
	2	0.9	**5**	63.59	**7**	-100.641
	3	0.889	11	58.79	1356	-35.565
	4	0.887	**6**	62.86	**8**	-100.329
	5	0.885	14	58.43	678	-54.0966
	6	0.883	**4**	64.02	**9**	-99.9212
	7	0.882	19	55.54	18	-92.4467
	8	0.871	**3**	66.11	**2**	-116.05
	9	0.862	17	56.25	17	-92.4525
	10	0.859	**8**	60.01	227	-70.3277
AvrPikD-PikmHMA (6fu9CD)	1	0.941	**3**	62.21	14	-100.436
	2	0.915	**1**	63.39	10	-104.785
	3	0.91	**2**	62.8	9	-104.797
	4	0.91	**4**	61.31	**2**	-111.478
	5	0.9	65	49.26	17	-98.704
	6	0.895	13	55.59	**3**	-110.116
	7	0.894	34	51.5	27	-93.2712
	8	0.861	90	47.9	55	-85.0911
	9	0.856	66	49.23	605	-54.2401
	10	0.852	54	49.91	428	-60.3741
AvrPikE-PikmHMA (6fubAB)	1	0.935	**4**	58.9	15	-90.305
	2	0.925	**1**	64.49	94	-76.1576
	3	0.888	**9**	55.74	34	-83.4692
	4	0.879	13	53.73	**3**	-102.602
	5	0.874	**6**	56.82	1084	-42.5602
	6	0.867	51	48.08	551	-55.3972
	7	0.867	17	51.68	143	-71.2714
	8	0.853	121	44.48	796	-49.6282
	9	0.852	**8**	56.06	698	-51.793
	10	0.849	50	48.12	930	-46.3857
AvrPikE-PikpHMA (6g11BC)	1	0.908	29	51.14	31	-76.3809
	2	0.899	22	53.06	385	-54.6336
	3	0.892	47	48.72	20	-77.9749
	4	0.892	**3**	62.15	**1**	-110.703
	5	0.885	**1**	65.69	**2**	-98.6024
	6	0.884	**4**	60.62	**3**	-95.3788
	7	0.875	**5**	60.16	65	-71.5955
	8	0.865	16	55.34	**4**	-94.4781
	9	0.864	27	51.3	**6**	-88.4164
	10	0.85	15	55.88	33	-75.8961
	1	0.947	**4**	58.82	22	-79.1564

AvrPikE-PikpHMA (6g11EF)	2	0.92	**9**	55.12	76	-70.4429
	3	0.897	**10**	54.72	43	-74.0056
	4	0.893	16	52.35	**2**	-93.2629
	5	0.879	**2**	60.49	12	-81.8776
	6	0.875	**6**	58.05	**1**	-96.3179
	7	0.869	19	51.26	**7**	-83.8453
	8	0.865	**3**	59.73	**6**	-84.8633
	9	0.853	122	44.01	653	-47.2859
	10	0.852	14	52.95	352	-55.9582
AvrPikE-PikpHMA mut (6r8mFG)	1	0.924	**2**	63.82	254	-61.0644
	2	0.901	**1**	67.9	**2**	-102.771
	3	0.871	**5**	59.85	**7**	-88.5242
	4	0.87	**10**	56.21	768	-44.7472
	5	0.857	**9**	57.54	1369	-27.2931
	6	0.824	**4**	61.54	63	-72.5668
	7	0.822	59	45.79	571	-50.0234
	8	0.819	16	54.36	458	-53.8054
	9	0.818	25	51.15	160	-65.0824
	10	0.815	44	47.02	132	-66.8959
AvrPikF-OsHIPP19 (7b1lBC)	1	0.928	**9**	63.75	94	-66.7719
	2	0.917	11	61.41	985	-32.7192
	3	0.914	12	61.17	**9**	-94.1515
	4	0.914	**2**	72.73	**8**	-95.1279
	5	0.907	**3**	72.57	88	-67.3141
	6	0.899	**6**	68.84	**6**	-97.2762
	7	0.887	**8**	65.44	14	-87.9087
	8	0.887	**1**	75.98	**3**	-111.826
	9	0.885	7	65.45	**10**	-90.3786
	10	0.88	**4**	70.92	**1**	-125.125
ApikL2F-sHMA94 (7nmmAI)	1	0.952	**3**	62.99	**1**	-103.174
	2	0.922	**1**	68.51	**9**	-91.4234
	3	0.921	**6**	59.51	106	-73.1485
	4	0.918	**2**	63.37	290	-63.4232
	5	0.896	23	50.8	180	-68.3114
	6	0.885	**7**	57.68	616	-51.7256
	7	0.87	12	53.89	**4**	-97.8778
	8	0.869	27	50.17	71	-76.8299
	9	0.864	**4**	61.76	123	-71.612
	10	0.863	**9**	56.02	178	-68.4282
ApikL2F-sHMA94 (7nmmBJ)	1	0.94	12	53.48	**1**	-102.48
	2	0.93	**8**	55.53	21	-88.9538
	3	0.924	**4**	57.62	159	-72.0868
	4	0.896	**5**	57.52	702	-49.6216

	5	0.888	11	53.48	17	-89.5989
	6	0.886	**3**	58.31	**9**	-93.9556
	7	0.885	42	47.7	287	-64.9626
	8	0.876	**1**	59.6	121	-74.7666
	9	0.863	23	49.9	248	-67.0649
	10	0.862	**7**	55.59	116	-75.0624
ApikL2F-sHMA94 (7nmmCK)	1	0.933	**3**	61.34	14	-88.5095
	2	0.911	**4**	60.91	**5**	-94.0781
	3	0.905	**5**	59.82	42	-79.243
	4	0.905	**2**	63.3	63	-76.7711
	5	0.898	16	53.23	22	-85.789
	6	0.886	19	52.07	**2**	-102.037
	7	0.877	**6**	58.48	19	-86.7354
	8	0.865	**8**	57.09	16	-87.847
	9	0.858	**1**	65.57	32	-81.8378
	10	0.857	28	48.02	278	-61.7858
ApikL2F-sHMA94 (7nmmDL)	1	0.943	**1**	70.17	**3**	-100.989
	2	0.931	**3**	61.26	**1**	-104.301
	3	0.884	10	57.09	16	-86.4131
	4	0.884	**2**	65.3	**2**	-103.054
	5	0.883	**9**	57.22	**8**	-92.0285
	6	0.873	**4**	60.17	29	-80.4307
	7	0.87	21	52.72	317	-58.4078
	8	0.868	**5**	58.63	13	-87.9235
	9	0.86	33	48.14	1747	-10.9675
	10	0.856	11	56.29	836	-42.1599
ApikL2F-sHMA94 (7nmmEM)	1	0.922	**8**	58.46	14	-89.2384
	2	0.922	5	59.95	16	-88.6768
	3	0.916	**2**	61.64	56	-80.4144
	4	0.903	**6**	58.92	**3**	-96.8506
	5	0.88	**7**	58.79	382	-61.876
	6	0.879	50	46.9	497	-58.291
	7	0.872	16	52.24	134	-73.4211
	8	0.87	**3**	61.38	112	-75.0739
	9	0.866	**9**	56.45	312	-64.4014
	10	0.864	12	54.21	262	-66.4954
ApikL2F-sHMA94 (7nmmFN)	1	0.933	**4**	61.17	**1**	-107.157
	2	0.922	**2**	64.53	**8**	-88.8082
	3	0.915	7	59.99	**2**	-104.589
	4	0.902	**10**	58.72	**4**	-98.4579
	5	0.898	**3**	64.2	17	-84.5845
	6	0.88	29	52.57	56	-77.5656
	7	0.874	20	55.39	185	-66.7935

	8	0.868	21	55.14	**6**	-89.579
	9	0.866	**1**	66.76	32	-80.8245
	10	0.866	12	57.8	233	-64.6182
ApikL2F-sHMA94 (7nmmGO)	1	0.941	**2**	64.19	**4**	-96.1059
	2	0.913	**1**	64.39	**6**	-93.2973
	3	0.91	11	54.59	15	-86.9455
	4	0.905	**8**	57.64	34	-82.0189
	5	0.901	**3**	60.89	**1**	-107.128
	6	0.898	**4**	60.83	85	-73.1651
	7	0.873	26	50.55	440	-55.2597
	8	0.862	**7**	59.06	26	-84.1119
	9	0.861	353	41.62	**3**	-96.3148
	10	0.858	16	53.04	508	-53.0032
ApikL2F-sHMA94 (7nmmHP)	1	0.914	**8**	57.23	**10**	-91.2643
	2	0.911	**9**	56.77	**9**	-92.0384
	3	0.91	**4**	58.29	21	-84.4108
	4	0.893	**5**	57.96	15	-89.2407
	5	0.89	**1**	63.64	**7**	-93.1513
	6	0.884	**7**	57.27	**2**	-98.7045
	7	0.87	21	51.08	274	-63.1149
	8	0.865	43	48.02	865	-44.0054
	9	0.863	62	46.61	123	-71.6632
	10	0.854	**3**	60.5	216	-66.0752

Table S3. Rankings of the top 10 best ZDOCK score docking poses and corresponding rankings using ZRANK score. Rankings shown in bold font indicate a top-10 pose for the ZRANK scoring function.

Effector-host receptor binding partner complex	ZDOCK ranking	ZDOCK score	ZRANK ranking	ZRANK score
Avr1CO39-RGA5 (5zngAC)	1	46.35	1261	-33.835
	2	45.52	1846	-5.42654
	3	45	1181	-36.3694
	4	44.56	82	-69.8359
	5	44.41	1286	-32.9555
	6	43.95	16	-78.0114
	7	43.63	111	-67.9328
	8	43.55	1546	-23.3259
	9	43.37	577	-51.716
	10	43.34	**2**	-87.4794
AvrPia-PikpHMA (6q76AB)	1	43.46	70	-73.1223
	2	43.26	125	-68.2589

	3	42.47	212	-64.0926
	4	42.21	1685	-19.1506
	5	41.29	263	-61.9065
	6	41.09	1285	-33.4787
	7	41.08	233	-63.227
	8	41.01	54	-74.507
	9	40.87	1144	-37.2376
	10	40.8	121	-68.4799
AvrPikC-PikhHMA (7a8xBC)	1	61.74	64	-70.0846
	2	60.16	**1**	-88.0183
	3	59.74	922	-38.9302
	4	58.01	**3**	-85.0714
	5	57.62	350	-55.8018
	6	57.58	**4**	-83.0148
	7	56.96	1079	-34.7113
	8	55.18	326	-56.894
	9	54.67	43	-72.5972
	10	54.5	1096	-34.3337
AvrPikC-PikhHMA (7a8xEF)	1	73.13	25	-85.0809
	2	69.6	22	-85.3532
	3	65.65	**5**	-102.248
	4	62.24	11	-90.3229
	5	62.03	43	-79.4627
	6	61.31	14	-88.0547
	7	60.47	**3**	-105.705
	8	60.38	**1**	-109.556
	9	59.42	1900	10.94890
	10	59.34	**2**	-108.377
AvrPikD-PikmHMA (6fu9AB)	1	69.34	**1**	-120.033
	2	66.8	14	-93.8471
	3	66.11	**2**	-116.050
	4	64.02	**9**	-99.9212
	5	63.59	**7**	-100.641
	6	62.86	**8**	-100.329
	7	61.22	**6**	-102.831
	8	60.01	227	-70.3277
	9	59.69	19	-92.2094
	10	59.45	177	-72.4639
AvrPikD-PikmHMA (6fu9CD)	1	63.39	**10**	-104.785
	2	62.8	**9**	-104.797
	3	62.21	14	-100.436
	4	61.31	**2**	-111.478
	5	60.53	**1**	-129.325

	6	58.13	31	-92.6532
	7	57.68	396	-61.5577
	8	57.54	38	-90.1360
	9	57.36	1095	-40.2118
	10	57.31	39	-89.5434
AvrPikE-PikmHMA (6fubAB)	1	64.49	94	-76.1576
	2	60.03	**2**	-103.783
	3	59.47	64	-79.4466
	4	58.9	15	-90.3050
	5	57.28	28	-85.7743
	6	56.82	1084	-42.5602
	7	56.65	22	-88.2068
	8	56.06	698	-51.7930
	9	55.74	34	-83.4692
	10	55.55	73	-78.3791
AvrPikE-PikpHMA (6g11BC)	1	65.69	**2**	-98.6024
	2	64.4	23	-77.5488
	3	62.15	**1**	-110.703
	4	60.62	**3**	-95.3788
	5	60.16	65	-71.5955
	6	58.87	**5**	-88.5445
	7	58.64	78	-70.1489
	8	58.48	75	-70.9139
	9	58.04	190	-62.1193
	10	57.82	251	-59.3259
AvrPikE-PikpHMA (6g11EF)	1	62.28	853	-42.1942
	2	60.49	12	-81.8776
	3	59.73	**6**	-84.8633
	4	58.82	22	-79.1564
	5	58.55	175	-63.8927
	6	58.05	**1**	-96.3179
	7	57.9	214	-61.6217
	8	55.95	143	-65.2232
	9	55.12	76	-70.4429
	10	54.72	43	-74.0056
AvrPikE-PikpHMA mut (6r8mFG)	1	67.9	**2**	-102.771
	2	63.82	254	-61.0644
	3	62.3	33	-78.3967
	4	61.54	63	-72.5668
	5	59.85	**7**	-88.5242
	6	59.17	453	-54.0219
	7	58.72	**3**	-91.6427
	8	58.28	43	-76.0868

	9	57.54	1369	-27.2931
	10	56.21	768	-44.7472
AvrPikF-OsHIPP19 (7b1lBC)	1	75.98	**3**	-111.826
	2	72.73	**8**	-95.1279
	3	72.57	88	-67.3141
	4	70.92	**1**	-125.125
	5	69.6	**4**	-105.916
	6	68.84	**6**	-97.2762
	7	65.45	**10**	-90.3786
	8	65.44	14	-87.9087
	9	63.75	94	-66.7719
	10	62.9	**2**	-112.413
ApikL2F-sHMA94 (7nmmAI)	1	68.51	**9**	-91.4234
	2	63.37	290	-63.4232
	3	62.99	**1**	-103.174
	4	61.76	123	-71.6120
	5	59.76	621	-51.5530
	6	59.51	106	-73.1485
	7	57.68	616	-51.7256
	8	56.23	182	-68.1792
	9	56.02	178	-68.4282
	10	55.94	346	-61.0129
ApikL2F-sHMA94 (7nmmBJ)	1	59.6	121	-74.7666
	2	58.69	102	-75.7036
	3	58.31	**9**	-93.9556
	4	57.62	159	-72.0868
	5	57.52	702	-49.6216
	6	57.21	**5**	-96.3640
	7	55.59	116	-75.0624
	8	55.53	21	-88.9538
	9	55.13	461	-57.6184
	10	55.13	1181	-34.8999
ApikL2F-sHMA94 (7nmmCK)	1	65.57	32	-81.8378
	2	63.3	63	-76.7711
	3	61.34	14	-88.5095
	4	60.91	**5**	-94.0781
	5	59.82	42	-79.2430
	6	58.48	19	-86.7354
	7	57.27	339	-58.4822
	8	57.09	16	-87.8470
	9	56.93	179	-66.8267
	10	56.02	13	-88.5285
	1	70.17	**3**	-100.989

ApikL2F-sHMA94 (7nmmDL)	2	65.3	**2**	-103.054
	3	61.26	**1**	-104.301
	4	60.17	29	-80.4307
	5	58.63	13	-87.9235
	6	57.91	1126	-34.4374
	7	57.83	60	-74.9995
	8	57.55	31	-79.9196
	9	57.22	**8**	-92.0285
	10	57.09	16	-86.4131
ApikL2F-sHMA94 (7nmmEM)	1	62.04	888	-47.3998
	2	61.64	56	-80.4144
	3	61.38	112	-75.0739
	4	61.2	36	-82.9554
	5	59.95	16	-88.6768
	6	58.92	**3**	-96.8506
	7	58.79	382	-61.8760
	8	58.46	14	-89.2384
	9	56.45	312	-64.4014
	10	54.53	292	-65.0592
ApikL2F-sHMA94 (7nmmFN)	1	66.76	32	-80.8245
	2	64.53	**8**	-88.8082
	3	64.2	17	-84.5845
	4	61.17	**1**	-107.157
	5	61.13	1574	-18.5380
	6	60.41	100	-72.6072
	7	59.99	**2**	-104.589
	8	59.77	344	-60.0000
	9	58.97	64	-75.9239
	10	58.72	**4**	-98.4579
ApikL2F-sHMA94 (7nmmGO)	1	64.39	**6**	-93.2973
	2	64.19	**4**	-96.1059
	3	60.89	**1**	-107.128
	4	60.83	85	-73.1651
	5	59.7	388	-56.9657
	6	59.64	222	-63.7676
	7	59.06	26	-84.1119
	8	57.64	34	-82.0189
	9	57.32	181	-65.6973
	10	55.43	158	-66.9573
ApikL2F-sHMA94 (7nmmHP)	1	63.64	**7**	-93.1513
	2	60.83	13	-90.0223
	3	60.5	216	-66.0752
	4	58.29	21	-84.4108

	5	57.96	15	-89.2407
	6	57.51	658	-50.6394
	7	57.27	**2**	-98.7045
	8	57.23	**10**	-91.2643
	9	56.77	**9**	-92.0384
	10	56.68	1582	-19.0348

Table S4. Rankings of the top 15 best ZRANK score docking poses and corresponding rankings based on ZDOCK and DockQ score. Docking poses with DockQ score above 0.8 are highlighted in bold, and docking poses omitted during application of residue restraints are shown in red font and italicised.

Effector-host receptor binding partner complex	ZRANK ranking	ZRANK score	ZDOCK ranking	ZDOCK score	DockQ ranking	DockQ score
Avr1CO39-RGA5 (5zngAC)	1	-89.5292	43	39.69	8	0.912
	2	-87.4794	10	43.34	59	0.655
	3	-86.2310	66	38.77	50	0.696
	4	-85.0222	198	36.81	689	0.079
	5	-84.5496	114	37.75	44	0.731
	6	*-83.1375*	*51*	*39.08*	*793*	*0.070*
	7	-82.4553	438	35.27	91	0.511
	8	-82.3644	1817	32.43	102	0.451
	9	-82.1716	279	36.14	72	0.594
	10	*-81.7161*	*787*	*34.11*	*573*	*0.086*
	11	-81.3406	734	34.25	39	0.753
	**12**	**-79.4734**	**103**	**38.01**	**29**	**0.807**
	13	*-79.4532*	*1747*	*32.52*	*251*	*0.130*
	14	-79.1197	42	39.92	56	0.675
	15	*-78.8399*	*1323*	*33.09*	*257*	*0.129*
AvrPia-PikpHMA (6q76AB)	*1*	*-95.9139*	*21*	*40.07*	*204*	*0.240*
	2	-91.2624	232	36.15	39	0.692
	3	*-89.1110*	*43*	*39.15*	*1677*	*0.023*
	4	*-88.7483*	*26*	*39.91*	*845*	*0.061*
	5	-87.7190	276	35.75	94	0.419
	6	*-86.4957*	*52*	*38.84*	*1600*	*0.024*
	7	-86.3842	83	38.11	54	0.597
	**8**	**-85.9788**	**579**	**34.09**	**25**	**0.821**
	9	*-84.8230*	*51*	*38.87*	*843*	*0.061*
	10	-84.7666	344	35.21	62	0.560
	11	*-84.6149*	*46*	*39.02*	*1601*	*0.024*

	12	*-83.7763*	*60*	*38.61*	*178*	*0.255*
	13	-83.7379	1852	31.68	41	0.674
	14	*-83.6471*	*533*	*34.31*	*777*	*0.065*
	15	*-83.0385*	*105*	*37.69*	*842*	*0.061*
AvrPikC-PikhHMA (7a8xBC)	**1**	**-88.0183**	**2**	**60.16**	**1**	**0.917**
	**2**	**-86.0303**	**20**	**49.02**	**18**	**0.808**
	**3**	**-85.0714**	**4**	**58.01**	**21**	**0.802**
	**4**	**-83.0148**	**6**	**57.58**	**8**	**0.878**
	5	*-82.1053*	*286*	*39.52*	*685*	*0.072*
	6	*-81.7334*	*1139*	*35.9*	*163*	*0.314*
	7	-81.7011	295	39.46	45	0.716
	8	*-81.6544*	*40*	*45.74*	*1858*	*0.017*
	9	*-81.6427*	*230*	*40.15*	*1644*	*0.018*
	10	*-81.6281*	*165*	*41.1*	*106*	*0.562*
	11	-80.7621	42	45.4	40	0.745
	12	*-80.6936*	*305*	*39.38*	*331*	*0.102*
	**13**	**-79.7873**	**11**	**53.75**	**3**	**0.888**
	**14**	**-79.3699**	**15**	**50.72**	**10**	**0.871**
	**15**	**-79.2047**	**31**	**47.28**	**25**	**0.788**
AvrPikC-PikhHMA (7a8xEF)	1	-109.5560	8	60.38	4	0.904
	2	-108.3770	10	59.34	16	0.831
	3	-105.7050	7	60.47	19	0.828
	4	-102.3670	76	47.7	43	0.731
	5	-102.2480	3	65.65	14	0.846
	6	*-95.5699*	*50*	*49.58*	*912*	*0.061*
	7	-93.1446	98	46.27	23	0.818
	8	-92.0097	46	49.91	29	0.784
	9	-91.7464	124	45.33	52	0.712
	10	-91.1429	14	58.45	11	0.854
	11	-90.3229	4	62.24	5	0.888
	12	*-90.2887*	*48*	*49.69*	*766*	*0.070*
	13	*-88.6367*	*21*	*54.18*	*375*	*0.103*
	14	-88.0547	6	61.31	24	0.813
	15	-87.4388	29	51.88	49	0.716
AvrPikD-PikmHMA (6fu9AB)	**1**	**-120.0330**	**1**	**69.34**	**13**	**0.833**
	**2**	**-116.0500**	**3**	**66.11**	**8**	**0.871**
	3	-106.4060	13	58.59	54	0.702
	4	-104.8450	29	52.52	49	0.717
	5	*-104.3320*	*141*	*44.31*	*349*	*0.119*
	**6**	**-102.8310**	**7**	**61.22**	**22**	**0.808**
	**7**	**-100.6410**	**5**	**63.59**	**2**	**0.900**
	**8**	**-100.3290**	**6**	**62.86**	**4**	**0.887**
	**9**	**-99.9212**	**4**	**64.02**	**6**	**0.883**

	10	*-99.6194*	*188*	*42.93*	*369*	*0.112*
	11	-99.2636	74	47.57	27	0.777
	12	*-97.7333*	*931*	*36.94*	*333*	*0.133*
	13	*-94.7468*	*163*	*43.54*	*358*	*0.116*
	**14**	**-93.8471**	**2**	**66.8**	**12**	**0.837**
	15	*-93.2170*	*364*	*40.06*	*598*	*0.089*
AvrPikD-PikmHMA (6fu9CD)	*1*	*-129.3250*	*5*	*60.53*	*296*	*0.120*
	**2**	**-111.4780**	**4**	**61.31**	**4**	**0.910**
	**3**	**-110.1160**	**13**	**55.59**	**6**	**0.895**
	4	*-109.7180*	*35*	*51.48*	*302*	*0.119*
	**5**	**-109.2430**	**41**	**51.17**	**14**	**0.842**
	6	*-107.6510*	*18*	*54.08*	*322*	*0.115*
	7	*-107.3680*	*57*	*49.79*	*316*	*0.116*
	8	*-105.6560*	*105*	*47.38*	*499*	*0.096*
	**9**	**-104.7970**	**2**	**62.8**	**3**	**0.910**
	**10**	**-104.7850**	**1**	**63.39**	**2**	**0.915**
	11	*-103.2640*	*1074*	*38.21*	*1380*	*0.023*
	12	*-103.0090*	*89*	*47.97*	*345*	*0.111*
	13	*-101.9700*	*126*	*46.45*	*364*	*0.108*
	**14**	**-100.4360**	**3**	**62.21**	**1**	**0.941**
	15	*-99.8241*	*394*	*41.88*	*999*	*0.056*
AvrPikE-PikmHMA (6fubAB)	1	-106.1790	554	39.73	239	0.162
	**2**	**-103.7830**	**2**	**60.03**	**13**	**0.838**
	**3**	**-102.6020**	**13**	**53.73**	**4**	**0.879**
	4	*-98.0256*	*239*	*42.3*	*1180*	*0.019*
	5	*-97.5260*	*127*	*44.35*	*177*	*0.231*
	6	*-95.8269*	*94*	*45.51*	*191*	*0.208*
	7	*-94.2026*	*130*	*44.27*	*358*	*0.095*
	8	*-93.5791*	*39*	*48.71*	*180*	*0.223*
	9	*-93.4517*	*1069*	*37.91*	*76*	*0.609*
	10	*-92.3534*	*628*	*39.35*	*1311*	*0.018*
	11	*-91.8758*	*479*	*40.13*	*186*	*0.210*
	12	*-91.4581*	*473*	*40.19*	*536*	*0.075*
	13	*-91.4434*	*177*	*43.51*	*283*	*0.119*
	14	-90.7172	26	50.08	22	0.798
	**15**	**-90.3050**	**4**	**58.9**	**1**	**0.935**
AvrPikE-PikpHMA (6g11BC)	**1**	**-110.7030**	**3**	**62.15**	**4**	**0.892**
	**2**	**-98.6024**	**1**	**65.69**	**5**	**0.885**
	**3**	**-95.3788**	**4**	**60.62**	**6**	**0.884**
	**4**	**-94.4781**	**16**	**55.34**	**8**	**0.865**
	**5**	**-88.5445**	**6**	**58.87**	**14**	**0.834**
	**6**	**-88.4164**	**27**	**51.3**	**9**	**0.864**
	7	*-88.2260*	*794*	*38.08*	*1613*	*0.018*

	8	*-86.0721*	*500*	*39.52*	*77*	*0.640*
	9	*-85.0710*	*799*	*38.07*	*606*	*0.090*
	10	*-84.8220*	*135*	*44.19*	*1977*	*0.015*
	11	-84.7155	17	55.11	37	0.743
	12	*-84.6581*	*522*	*39.44*	*860*	*0.067*
	13	*-84.1951*	*34*	*50.42*	*1984*	*0.015*
	14	*-83.2916*	*1100*	*37.06*	*613*	*0.089*
	15	*-82.2992*	*138*	*44.14*	*535*	*0.096*
AvrPikE-PikpHMA (6g11EF)	**1**	**-96.3179**	**6**	**58.05**	**6**	**0.875**
	**2**	**-93.2629**	**16**	**52.35**	**4**	**0.893**
	3	*-93.0332*	*677*	*38.62*	*1754*	*0.017*
	4	*-89.4946*	*143*	*43.33*	*576*	*0.093*
	5	*-88.4454*	*755*	*38.35*	*251*	*0.136*
	**6**	**-84.8633**	**3**	**59.73**	**8**	**0.865**
	**7**	**-83.8453**	**19**	**51.26**	**7**	**0.869**
	8	*-83.0799*	*365*	*40.51*	*159*	*0.335*
	9	*-83.0183*	*496*	*39.55*	*622*	*0.089*
	10	*-82.0344*	*1635*	*36.03*	*96*	*0.580*
	11	*-81.8788*	*277*	*41.23*	*603*	*0.091*
	**12**	**-81.8776**	**2**	**60.49**	**5**	**0.879**
	13	*-81.5497*	*264*	*41.36*	*832*	*0.069*
	14	*-81.3722*	*616*	*38.92*	*829*	*0.069*
	15	*-81.2861*	*353*	*40.57*	*757*	*0.076*
AvrPikE-PikpHMA mut (6r8mFG)	**1**	**-112.6550**	**30**	**50.5**	**13**	**0.807**
	**2**	**-102.7710**	**1**	**67.9**	**2**	**0.901**
	3	-91.6427	7	58.72	34	0.733
	4	*-89.3933*	*661*	*37.55*	*940*	*0.055*
	5	*-88.8784*	*607*	*37.88*	*66*	*0.664*
	6	-88.5705	17	54.35	27	0.769
	**7**	**-88.5242**	**5**	**59.85**	**3**	**0.871**
	8	*-87.9761*	*498*	*38.44*	*149*	*0.464*
	9	*-86.6719*	*74*	*45.13*	*152*	*0.457*
	10	-86.5235	14	54.55	39	0.713
	11	-86.3998	585	37.96	234	0.151
	12	-86.1257	490	38.47	332	0.105
	13	-84.9338	116	43	64	0.667
	14	*-84.8296*	*158*	*41.96*	*327*	*0.106*
	15	*-84.8253*	*769*	*37.16*	*296*	*0.110*
AvrPikF-OsHIPP19 (7b1lBC)	**1**	**-125.1250**	**4**	**70.92**	**10**	**0.880**
	**2**	**-112.4130**	**10**	**62.9**	**17**	**0.839**
	**3**	**-111.8260**	**1**	**75.98**	**8**	**0.887**
	**4**	**-105.9160**	**5**	**69.6**	**11**	**0.876**
	**5**	**-99.3500**	**20**	**58.62**	**13**	**0.851**

	**6**	**-97.2762**	**6**	**68.84**	**6**	**0.899**
	**7**	**-96.2969**	**18**	**60.01**	**21**	**0.824**
	**8**	**-95.1279**	**2**	**72.73**	**4**	**0.914**
	**9**	**-94.1515**	**12**	**61.17**	**3**	**0.914**
	**10**	**-90.3786**	**7**	**65.45**	**9**	**0.885**
	11	-89.4731	28	53.42	44	0.749
	12	-89.3410	31	53.08	34	0.776
	13	-88.8375	32	52.59	50	0.740
	**14**	**-87.9087**	**8**	**65.44**	**7**	**0.887**
	15	-87.7319	38	51.26	32	0.789
pikL2A-sHMA94 (7nmmAI)	**1**	**-103.1740**	**3**	**62.99**	**1**	**0.952**
	2	*-98.5421*	*58*	*45.42*	*454*	*0.065*
	3	*-98.3968*	*1279*	*37.46*	*130*	*0.233*
	**4**	**-97.8778**	**12**	**53.89**	**7**	**0.870**
	5	*-96.3848*	*389*	*40.3*	*74*	*0.641*
	6	*-92.4402*	*53*	*45.8*	*466*	*0.064*
	7	*-91.9992*	*174*	*42.11*	*518*	*0.060*
	8	*-91.7799*	*909*	*38.35*	*656*	*0.050*
	**9**	**-91.4234**	**1**	**68.51**	**2**	**0.922**
	10	*-90.9236*	*739*	*38.79*	*424*	*0.068*
	11	*-90.4663*	*120*	*43.26*	*1741*	*0.018*
	12	*-90.4247*	*869*	*38.47*	*371*	*0.073*
	13	*-90.1579*	*64*	*44.98*	*498*	*0.062*
	14	*-89.9620*	*1527*	*37.09*	*313*	*0.078*
	15	*-87.8101*	*229*	*41.46*	*587*	*0.056*
pikL2A-sHMA94 (7nmmBJ)	**1**	**-102.4800**	**12**	**53.48**	**1**	**0.940**
	2	*-98.5070*	*1306*	*36.14*	*259*	*0.089*
	3	*-97.9469*	*896*	*37.17*	*554*	*0.061*
	4	*-96.5826*	*27*	*49.41*	*1805*	*0.017*
	**5**	**-96.3640**	**6**	**57.21**	**13**	**0.848**
	6	*-94.7932*	*618*	*38.17*	*381*	*0.079*
	7	*-94.1881*	*799*	*37.49*	*143*	*0.307*
	8	*-94.0107*	*641*	*38.06*	*603*	*0.058*
	**9**	**-93.9556**	**3**	**58.31**	**6**	**0.886**
	10	*-92.9697*	*1851*	*35.23*	*510*	*0.065*
	11	*-92.4721*	*80*	*44.67*	*607*	*0.058*
	12	*-92.1951*	*1809*	*35.3*	*168*	*0.225*
	13	*-91.4489*	*634*	*38.09*	*573*	*0.060*
	14	*-90.0133*	*289*	*40.69*	*643*	*0.056*
	15	*-89.8092*	*1966*	*35.09*	*221*	*0.095*
pikL2A-sHMA94 (7nmmCK)	**1**	**-102.3810**	**20**	**51.91**	**15**	**0.834**
	**2**	**-102.0370**	**19**	**52.07**	**6**	**0.886**
	3	*-101.8300*	*577*	*39.18*	*80*	*0.642*

	4	*-96.5480*	*283*	*40.81*	*440*	*0.070*
	**5**	**-94.0781**	**4**	**60.91**	**2**	**0.911**
	6	*-93.3125*	*147*	*42.35*	*483*	*0.064*
	7	*-92.2864*	*730*	*38.61*	*438*	*0.070*
	8	*-91.2961*	*1674*	*36.43*	*435*	*0.070*
	9	*-90.9599*	*1695*	*36.4*	*484*	*0.063*
	10	*-90.9374*	*97*	*43.48*	*524*	*0.060*
	11	*-89.8206*	*323*	*40.54*	*361*	*0.081*
	12	*-88.5927*	*866*	*38.15*	*965*	*0.032*
	**13**	**-88.5285**	**10**	**56.02**	**13**	**0.843**
	**14**	**-88.5095**	**3**	**61.34**	**1**	**0.933**
	15	*-88.0200*	*174*	*42.02*	*44*	*0.745*
pikL2A-sHMA94 (7nmmDL)	**1**	**-104.3010**	**3**	**61.26**	**2**	**0.931**
	**2**	**-103.0540**	**2**	**65.3**	**4**	**0.884**
	**3**	**-100.9890**	**1**	**70.17**	**1**	**0.943**
	4	*-98.6563*	*94*	*43.32*	*525*	*0.071*
	5	*-98.1414*	*816*	*37.34*	*353*	*0.087*
	6	*-97.3567*	*58*	*46.31*	*1748*	*0.018*
	7	*-93.3654*	*374*	*39.36*	*246*	*0.106*
	**8**	**-92.0285**	**9**	**57.22**	**5**	**0.883**
	9	*-90.9654*	*53*	*46.73*	*1833*	*0.017*
	10	*-89.3951*	*1152*	*36.56*	*531*	*0.069*
	11	*-88.6364*	*31*	*48.46*	*1835*	*0.017*
	12	*-88.1059*	*105*	*42.83*	*1832*	*0.017*
	**13**	**-87.9235**	**5**	**58.63**	**8**	**0.868**
	14	-87.2222	864	37.22	478	0.076
	15	-87.1608	1642	35.64	248	0.105
pikL2A-sHMA94 (7nmmEM)	*1*	*-98.7345*	*89*	*44.6*	*413*	*0.065*
	2	*-96.9769*	*44*	*47.52*	*443*	*0.062*
	**3**	**-96.8506**	**6**	**58.92**	**4**	**0.903**
	4	*-96.2425*	*76*	*45.19*	*131*	*0.101*
	5	*-94.7303*	*1242*	*37.32*	*365*	*0.068*
	6	*-92.5136*	*125*	*43.68*	*484*	*0.060*
	7	*-92.2882*	*1682*	*36.49*	*132*	*0.100*
	8	-92.0933	1433	36.89	62	0.617
	9	*-91.9472*	*225*	*41.77*	*1215*	*0.022*
	10	-90.8424	303	41.05	63	0.615
	11	*-90.7821*	*1535*	*36.7*	*119*	*0.104*
	12	*-90.2980*	*182*	*42.53*	*538*	*0.057*
	13	*-89.9076*	*245*	*41.62*	*340*	*0.071*
	**14**	**-89.2384**	**8**	**58.46**	**1**	**0.922**
	15	*-88.7354*	*1180*	*37.47*	*448*	*0.061*
	**1**	**-107.1570**	**4**	**61.17**	**1**	**0.933**

pikL2A-sHMA94 (7nmmFN)	**2**	**-104.5890**	**7**	**59.99**	**3**	**0.915**
	3	*-98.8737*	*34*	*49.94*	*324*	*0.086*
	**4**	**-98.4579**	**10**	**58.72**	**4**	**0.902**
	5	-94.6601	35	49.84	52	0.724
	**6**	**-89.5790**	**21**	**55.14**	**8**	**0.868**
	7	*-89.2866*	*801*	*38.89*	*673*	*0.052*
	**8**	**-88.8082**	**2**	**64.53**	**2**	**0.922**
	9	*-88.4652*	*1157*	*37.91*	*467*	*0.071*
	10	*-86.3931*	*515*	*40.06*	*1593*	*0.019*
	11	*-85.7750*	*1244*	*37.71*	*1038*	*0.031*
	12	*-85.6505*	*1351*	*37.51*	*531*	*0.064*
	13	*-85.3202*	*182*	*42.98*	*1247*	*0.024*
	14	-85.1810	24	54.39	35	0.769
	15	*-84.9025*	*597*	*39.67*	*1839*	*0.016*
pikL2A-sHMA94 (7nmmGO)	**1**	**-107.1280**	**3**	**60.89**	**5**	**0.901**
	**2**	**-97.7361**	**15**	**53.57**	**22**	**0.811**
	**3**	**-96.3148**	**353**	**41.62**	**9**	**0.861**
	**4**	**-96.1059**	**2**	**64.19**	**1**	**0.941**
	5	*-95.2097*	*148*	*44.23*	*286*	*0.084*
	**6**	**-93.2973**	**1**	**64.39**	**2**	**0.913**
	7	*-93.2249*	*607*	*40.07*	*432*	*0.067*
	8	*-90.2696*	*827*	*39.26*	*158*	*0.106*
	9	*-90.1811*	*190*	*43.38*	*12*	*0.845*
	10	*-89.6073*	*84*	*45.86*	*1598*	*0.017*
	11	*-89.0801*	*1371*	*37.82*	*369*	*0.076*
	12	*-89.0095*	*81*	*45.98*	*512*	*0.059*
	13	*-88.3740*	*889*	*39.05*	*243*	*0.089*
	14	*-87.6273*	*168*	*43.8*	*476*	*0.062*
	**15**	**-86.9455**	**11**	**54.59**	**3**	**0.910**
pikL2A-sHMA94 (7nmmHP)	*1*	*-103.7700*	*587*	*39.01*	*522*	*0.061*
	**2**	**-98.7045**	**7**	**57.27**	**6**	**0.884**
	3	*-96.3243*	*193*	*42.66*	*1614*	*0.018*
	4	*-94.6288*	*1109*	*37.24*	*968*	*0.030*
	**5**	**-94.3900**	**13**	**53.05**	**13**	**0.833**
	6	*-94.0671*	*1131*	*37.2*	*1989*	*0.013*
	**7**	**-93.1513**	**1**	**63.64**	**5**	**0.890**
	8	*-92.3917*	*848*	*38.01*	*1548*	*0.019*
	**9**	**-92.0384**	**9**	**56.77**	**2**	**0.911**
	**10**	**-91.2643**	**8**	**57.23**	**1**	**0.914**
	11	*-90.9757*	*75*	*45.99*	*1605*	*0.018*
	12	*-90.8388*	*129*	*44.3*	*597*	*0.054*
	**13**	**-90.0223**	**2**	**60.83**	**21**	**0.817**
	14	*-89.9810*	*1261*	*36.92*	*795*	*0.037*

	15	-89.2407	5	57.96	4	0.893

Table S5: List of fungal effector protein and their host receptor binding partners in MAX complexes, and important amino acid residues involved in the interactions between them as reported in the literature. Active residues are those involved in the interaction at the interface and passive residues are known not to be involved in the binding of fungal effector protein to the host partner.

**Effector and fungal species**	**PDB ID: Chains**	**Active and passive residues**	**Host receptor binding partner, plant species, PDB ID and active and passive residues**	**References**
Avr1CO39<br>*M. oryzae*	5ZNG*:C	Active:<br>*H-bonds*: Trp23, Lys24, Asn37, Ile39, Thr41<br>*Hydrophobic contacts*: Ala22, Ile27, Asn38, Tyr40	RGA5<br>*Oryza sativa*<br>(5ZNG*:A)<br><br>*Binding areas with Avr and dimerisation*:<br>*H-bonds*: Val1025, Asp1026, Val1028, Ile1030.<br>*Hydrophobic contacts*: Met1016, Ala1017, Ala1020, Ser1021, Asn1023, Gly1024, Ser1027, Glu1029	(Cesari et al., 2013; Guo et al., 2018)
AVR-Pia<br>*M. oryzae*	6Q76*:B	Active:<br>Leu38, Tyr41, Arg43, Tyr85	PikpHMA<br>*Oryza sativa*, *Nicotiana benthamiana*<br>(6Q76*:A)<br><br>*Binding residues:*<br>Ser204, Ser212, Asp217, Val219, Arg226	(Varden et al., 2019)
Avr-PikC<br>*M. oryzae*	7A8X*:C,F	Active:<br>*Binding interface:*<br>1: Thr69, Ile49<br>2: Asn46, Pro47, Gly48, Arg64, Asp66<br>3: Glu53, Tyr71, Ser72, Trp74	Pikh-HMA<br>(7A8X*:A,B,D,E)<br>*Oryza sativa*<br><br>*Binding interface*<br>1: Leu188-Lys191<br>2: Ser218-Val232<br>3: Glu253-Asp264	(De La Concepcion et al., 2021)
Avr-PikD<br>*M. oryzae*	6FU9*:B,D	Active:<br>*Binding interface:*<br>1: Thr69, Ile49<br>2: His46, Pro47, Gly48, Arg64, Asp66<br>3: Glu53, Ser72, Trp74	Pikm-HMA<br>*Oryza sativa*<br>(6FU9*: A,C)<br><br>*Binding interface:*<br>1: Glu188-Lys191<br>2: Ser219-Val233, Lys195<br>3: Met254-Asp264	(De La Concepcion et al., 2018)

Avr-PikE<br>*M. oryzae*	6G11*:C,F<br>6R8M*:G<br>6FUB*:B	Active:<br>*Binding interface:*<br>1: Thr69, Ile49<br>2: Asn46, Pro47, Gly48, Arg64, Asp66<br>3: Glu53, Ser72, Trp74	PikpHMA<br>*Oryza sativa,*<br>*Nicotiana benthamiana*<br>(6G11*: B,E)<br>Pikp -HMA^{NK-KE}(6R8M*:F)<br><br>*Binding interface:*<br>1: Leu188-Lys191<br>2: Ser218-Val232<br>3: Glu253-Asp264	(De La Concepcion et al., 2018, 2019)
		Active:<br>*Binding interface:*<br>1: Thr69, Ile49<br>2: Asn46, Pro47, Gly48, Arg64, Asp66<br>3: Glu53, Tyr71, Ser72, Trp74	Pikm-HMA<br>*Oryza sativa*<br>(6FUB*: A)<br><br>*Binding interface:*<br>1: Glu188-Lys191<br>2: Ser219-Val233, Lys195<br>3: Met254-Asp264	(De La Concepcion et al., 2018)
Avr-PikF<br>*M. oryzae*	7B1I*:C	Active:<br>*Binding interface:*<br>1: Thr69<br>2: Asn46, Ala67, Asp68<br>3: Glu53, Try71, Ser72, Trp74<br>Lys78	OsHIPP19<br>*Oryza sativa*<br>(7B1I*:B)<br><br>*Binding interface:*<br>1: Lys4<br>2: Ser31-Val46<br>3: Glu67-Glu76	(Maidment et al., 2021)
ApikL2F<br>*M. oryzae*	7NMM*:<br>A,B,C,D,E,F,<br>G,H	Active:<br>*Binding interface:*<br>1: Val69, Ile49<br>2: His46, Pro47, Gly48, Arg64, Asn66<br>3: Asp53, Ser72, Trp74	sHMA94<br>*Setaria italica*<br>(7NMM*: I,J,K,L,M,N,O,P)<br><br>*Binding interface:*<br>1: Leu5-Ile8<br>2: Ser33-Val47<br>3: Arg68-Glu76	(Bentham et al., 2021)

*Effector-plant protein complex structure.
**Numbered groups in active and passive residues represent residues that are known to specifically interact with the residues in the corresponding host receptor binding partner.

1. S. Cesari, G. Thilliez, C. Ribot, V. Chalvon, C. Michel, A. Jauneau, S. Rivas, L. Alaux, H. Kanzaki, Y. Okuyama, J. B. Morel, E. Fournier, D. Tharreau, R. Terauchi, T. Kroj, The rice resistance protein pair RGA4/RGA5 recognizes the Magnaporthe oryzae effectors AVR-Pia and AVR1-CO39 by direct binding, The Plant cell. 25 (2013) 1463–1481. https://doi.org/10.1105/tpc.112.107201.
2. L. Guo, S. Cesari, K. de Guillen, V. Chalvon, L. Mammri, M. Ma, I. Meusnier, F. Bonnot, A. Padilla, Y.L. Peng, J. Liu, T. Kroj, Specific recognition of two MAX effectors by integrated HMA domains in plant immune receptors involves distinct binding surfaces, Proceedings of the National Academy of Sciences of the United States of America. 115 (2018) 11637–11642. https://doi.org/10.1073/pnas.1810705115.
3. F.A. Varden, H. Saitoh, K. Yoshino, M. Franceschetti, S. Kamoun, R. Terauchi, M.J. Banfield, Cross-reactivity of a rice NLR immune receptor to distinct effectors from the rice blast pathogen

Magnaporthe oryzae provides partial disease resistance, The Journal of biological chemistry. 294 (2019) 13006–13016. https://doi.org/10.1074/jbc.RA119.007730.

4. J.C. De la Concepcion, J.H.R. Maidment, A. Longya, G. Xiao, M. Franceschetti, M.J. Banfield, The allelic rice immune receptor Pikh confers extended resistance to strains of the blast fungus through a single polymorphism in the effector binding interface, PLOS Pathog. 17 (2021) e1009368. https://doi.org/10.1371/journal.ppat.1009368.
5. J.C. De la Concepcion, M. Franceschetti, A. Maqbool, H. Saitoh, R. Terauchi, S. Kamoun, M.J. Banfield, Polymorphic residues in rice NLRs expand binding and response to effectors of the blast pathogen, Nature plants. 4 (2018) 576–585. https://doi.org/10.1038/s41477-018-0194-x.
6. J.C. De la Concepcion, M. Franceschetti, D. MacLean, R. Terauchi, S. Kamoun, M.J. Banfield, Protein engineering expands the effector recognition profile of a rice NLR immune receptor, eLife. 8 (2019) e47713. https://doi.org/10.7554/eLife.47713.
7. J.H. Maidment, M. Franceschetti, A. Maqbool, H. Saitoh, C. Jantasuriyarat, S. Kamoun, R. Terauchi, M.J. Banfield, Multiple variants of the fungal effector AVR-Pik bind the HMA domain of the rice protein OsHIPP19, providing a foundation to engineer plant defense, Journal of Biological Chemistry. 296 (2021). https://doi.org/10.1074/jbc.RA119.007730.
8. A.R. Bentham, Y. Petit-Houdenot, J. Win, I. Chuma, R. Terauchi, M.J. Banfield, S. Kamoun, T. Langner, A single amino acid polymorphism in a conserved effector of the multihost blast fungus pathogen expands host-target binding spectrum, PLoS pathogens. 17 (2021) e1009957. https://doi.org/10.1371/journal.ppat.1009957.

Figure S1. Correlation of ZDOCK score versus DockQ score for ZDOCK output of 54,000 docking poses for experimental complexes used in benchmarking. The docking pose with the best ZDOCK score is highlighted in red.

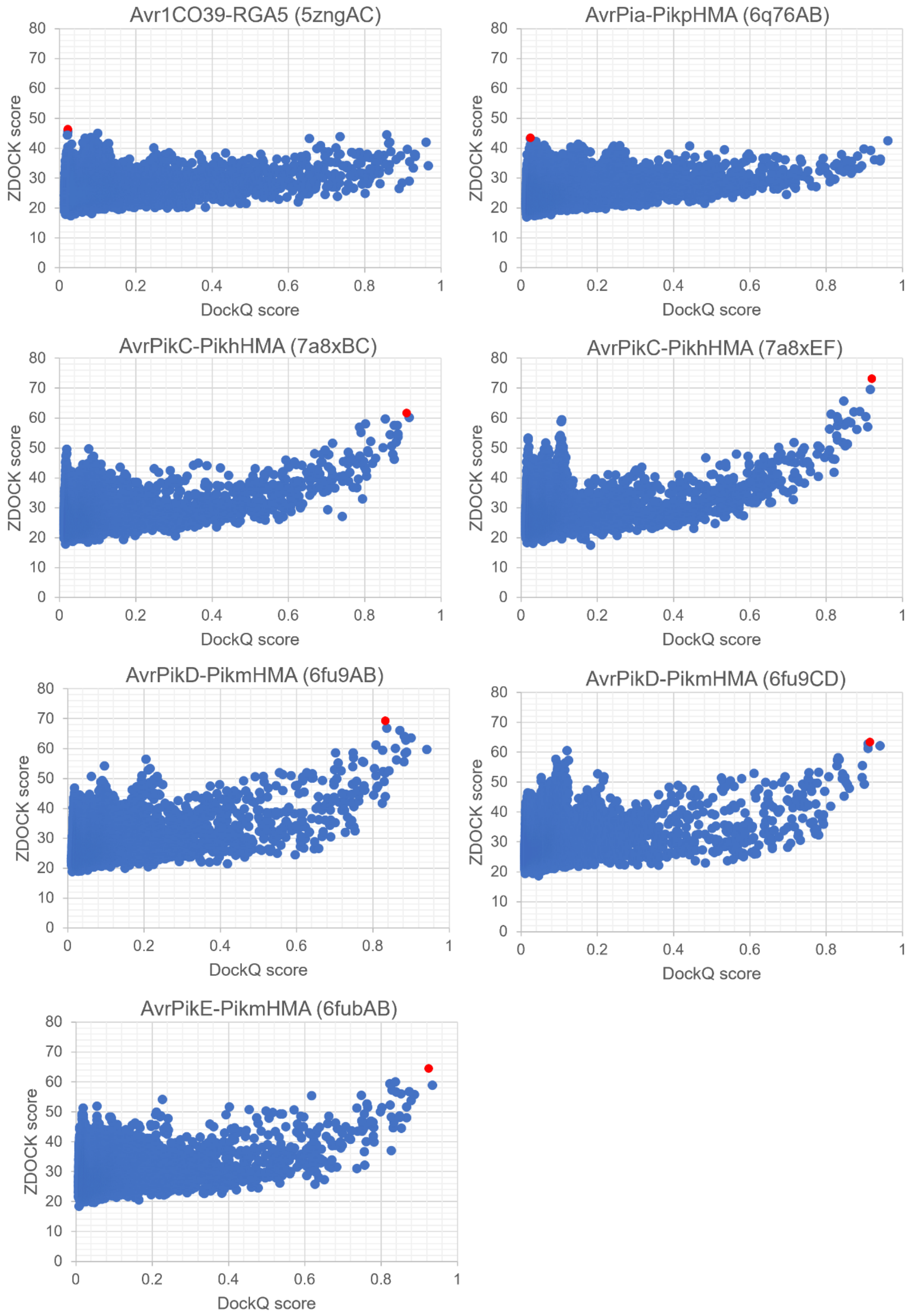

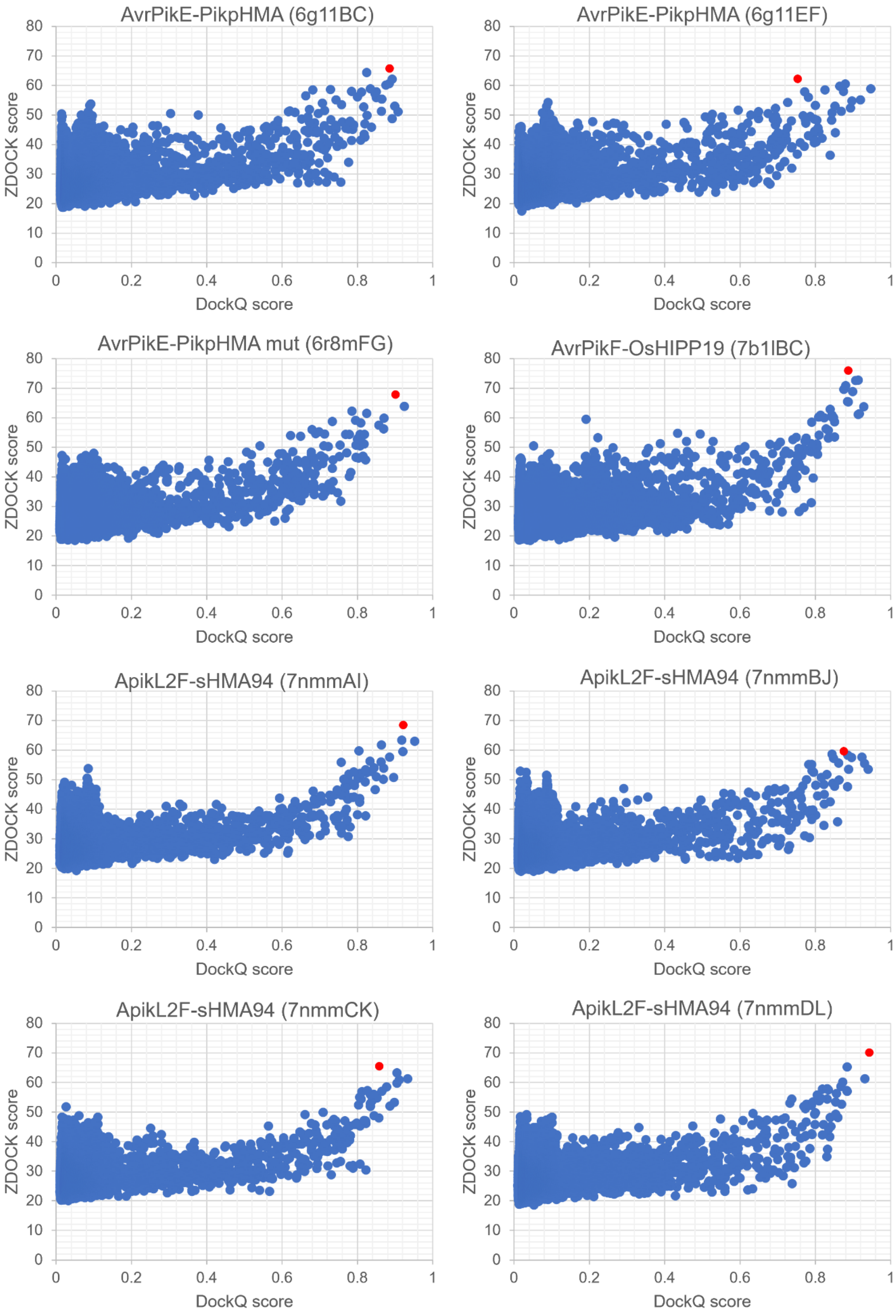
AvrPikE-PikpHMA (6g11BC)
ZDOCK score
DockQ score
AvrPikE-PikpHMA (6g11EF)
ZDOCK score
DockQ score
AvrPikE-PikpHMA mut (6r8mFG)
ZDOCK score
DockQ score
AvrPikF-OsHIPP19 (7b1IBC)
ZDOCK score
DockQ score
ApikL2F-sHMA94 (7nmmAI)
ZDOCK score
DockQ score
ApikL2F-sHMA94 (7nmmBJ)
ZDOCK score
DockQ score
ApikL2F-sHMA94 (7nmmCK)
ZDOCK score
DockQ score
ApikL2F-sHMA94 (7nmmDL)
ZDOCK score
DockQ score

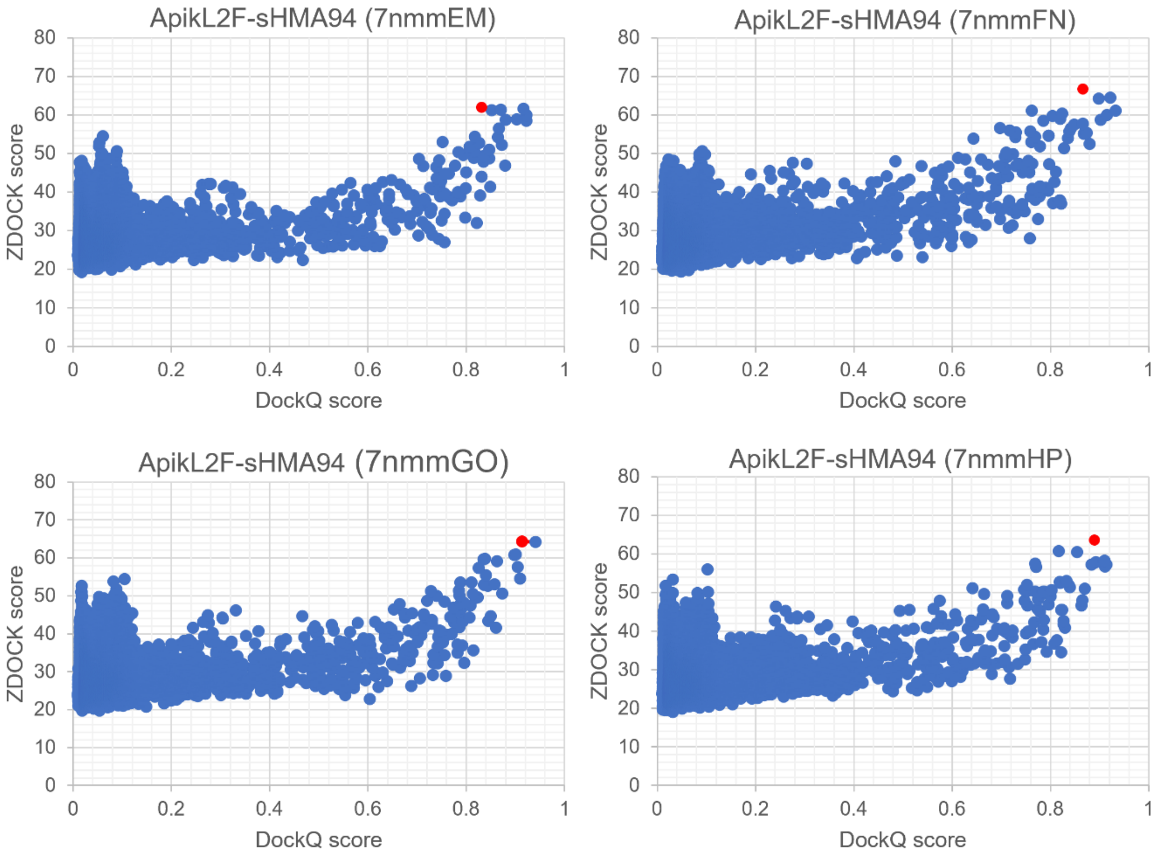

Figure S2. Correlation of ZRANK score versus DockQ score for the top 2000 ZDOCK score docking poses. Poses with the best ZRANK score are highlighted in red.

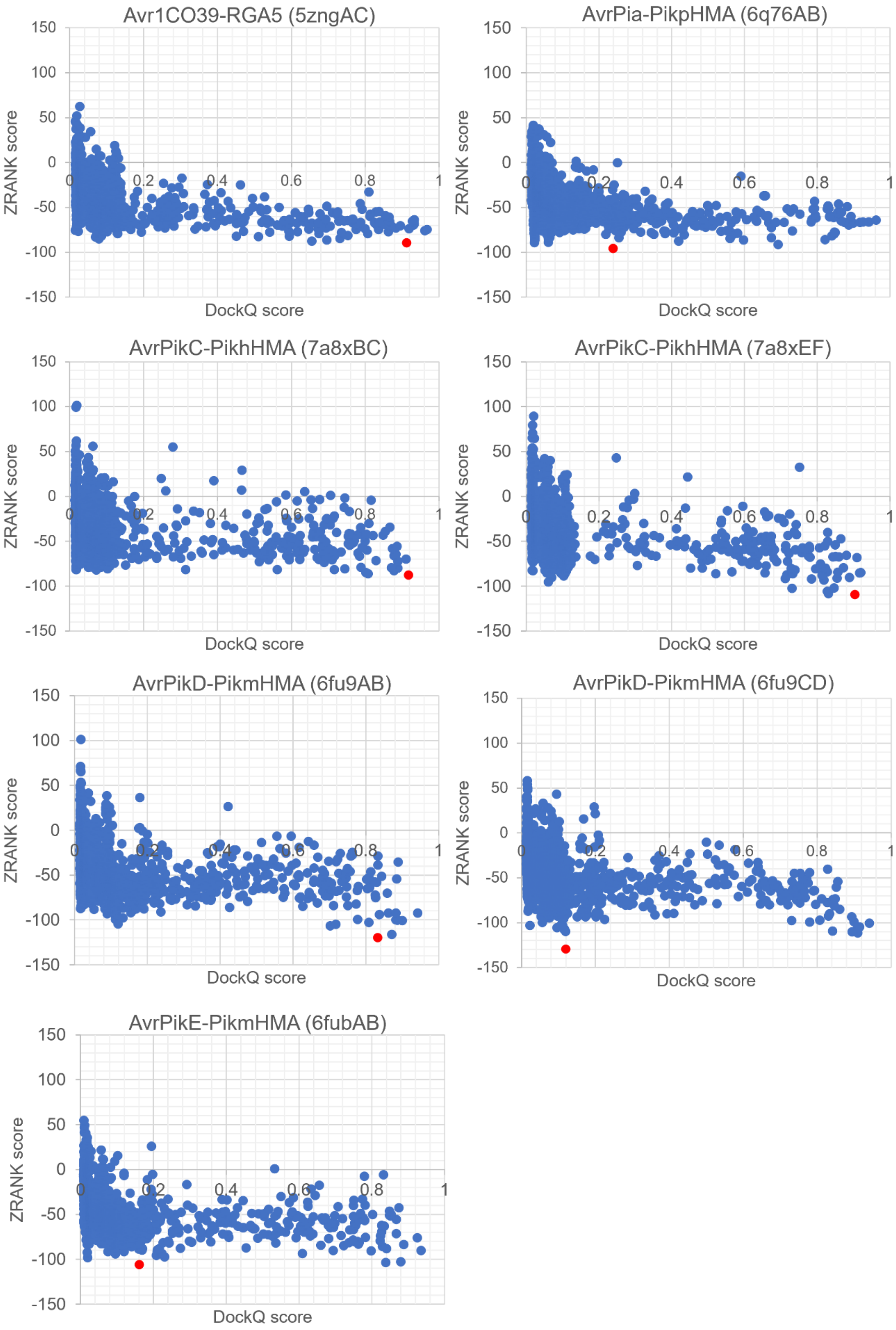
Avr1CO39-RGA5 (5zngAC)
AvrPia-PikpHMA (6q76AB)
AvrPikC-PikhHMA (7a8xBC)
AvrPikC-PikhHMA (7a8xEF)
AvrPikD-PikmHMA (6fu9AB)
AvrPikD-PikmHMA (6fu9CD)
AvrPikE-PikmHMA (6fubAB)
ZRANK score
DockQ score
150
100
50
0
-50
-100
-150
0
0.2
0.4
0.6
0.8
1

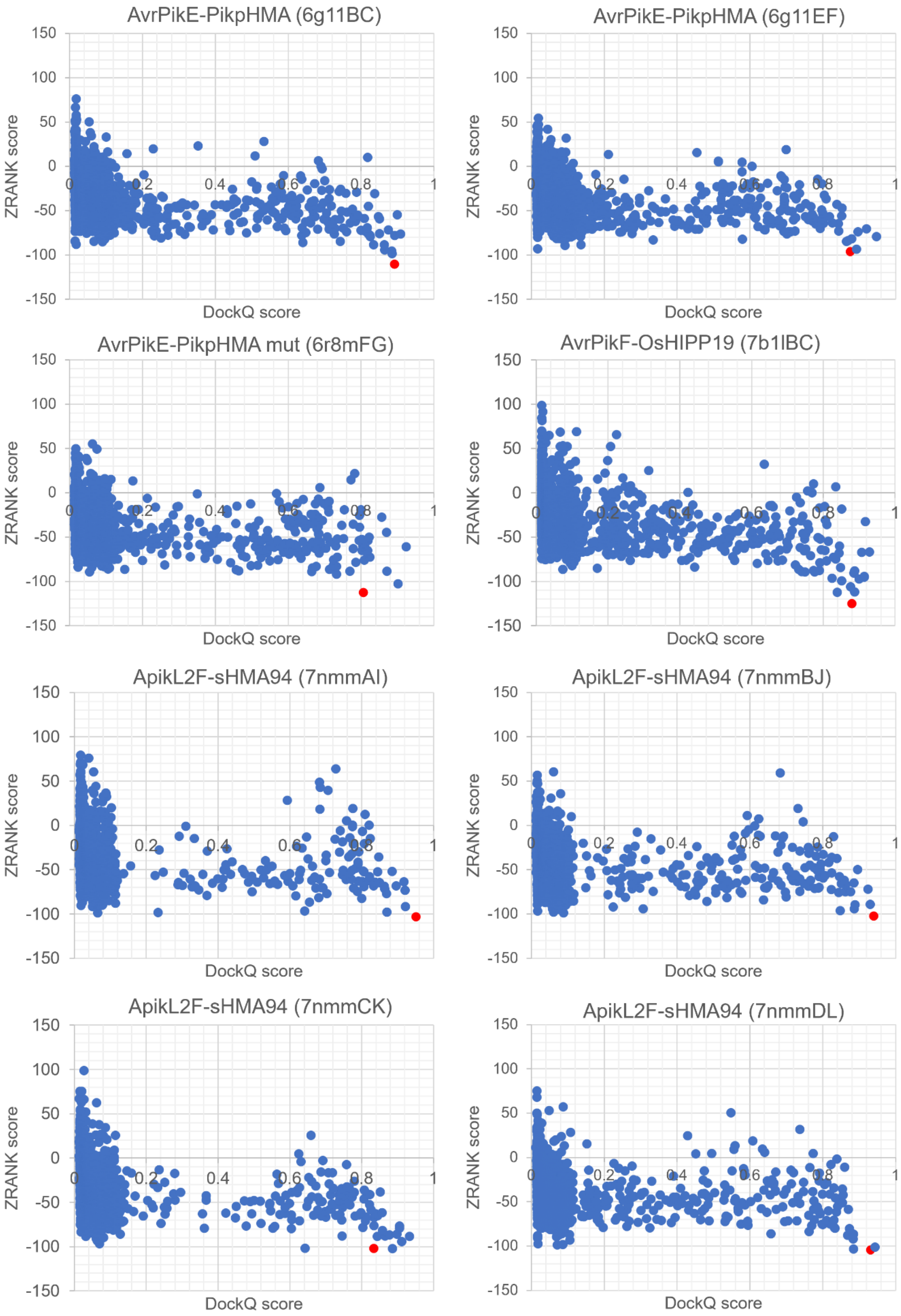
AvrPikE-PikpHMA (6g11BC)
AvrPikE-PikpHMA (6g11EF)
AvrPikE-PikpHMA mut (6r8mFG)
AvrPikF-OsHIPP19 (7b1lBC)
ApikL2F-sHMA94 (7nmmAI)
ApikL2F-sHMA94 (7nmmBJ)
ApikL2F-sHMA94 (7nmmCK)
ApikL2F-sHMA94 (7nmmDL)
ZRANK score
DockQ score
150
100
50
0
-50
-100
-150
0
0.2
0.4
0.6
0.8
1

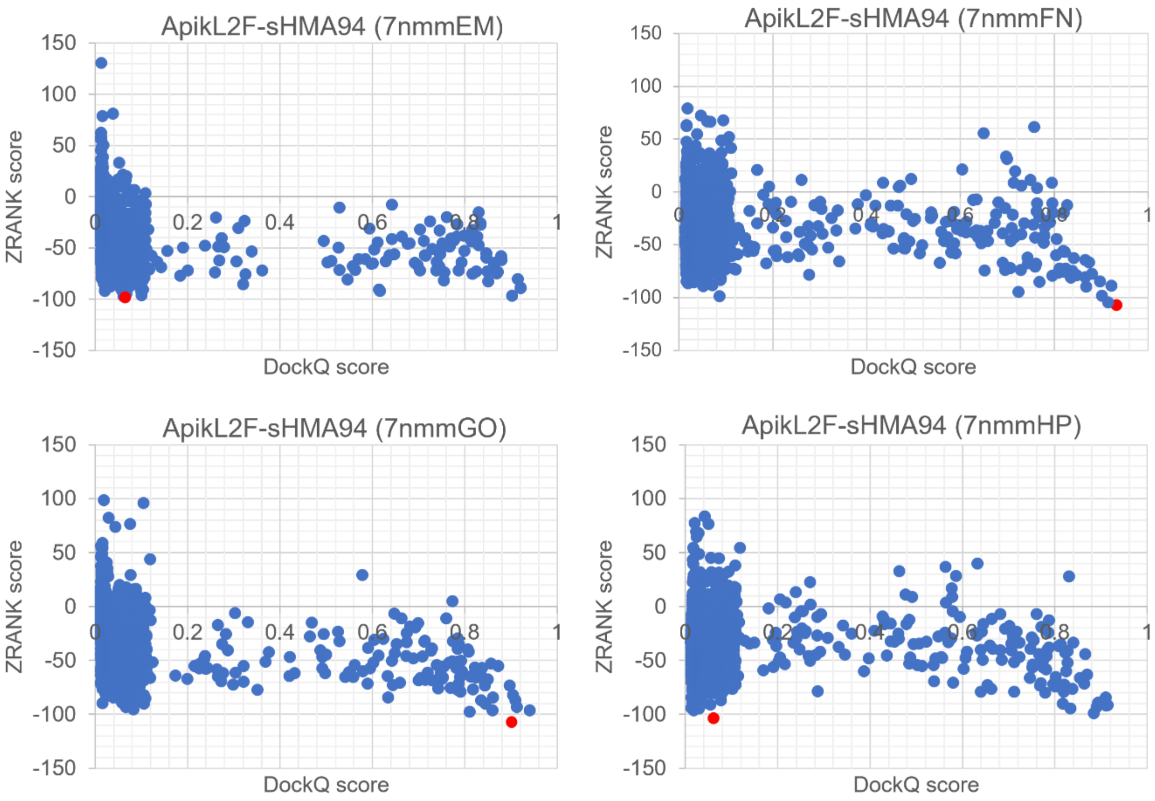
ApikL2F-sHMA94 (7nmmEM)
ApikL2F-sHMA94 (7nmmFN)
ApikL2F-sHMA94 (7nmmGO)
ApikL2F-sHMA94 (7nmmHP)
ZRANK score
DockQ score
150
100
50
0
-50
-100
-150
0
0.2
0.4
0.6
0.8
1

Assessment of docking output with and without residue restraints

Similar to blind docking, all docking poses were assigned a ZDOCK score, ZRANK score and DockQ score, and the position/ranking of near-native docking poses was determined. In this study, only active residues were implemented and due to this, differences in the predictions between both docking approaches (with and without residues restraints) were only observed after re-scoring and re-ranking with ZRANK, such that the rankings of the top 2000 ZDOCK docking poses were similar for both docking approaches. After ZRANK re-scoring, differences in the total number of ZRANK docking poses (out of 2,000) were observed due to the use of residue restraints, which omitted poses that do not fulfil the restrains (Figure S3). Plots for all other bound complexes are in Figure S4. Detailed information on the docking poses that were excluded within the top 15 docking poses ranked with ZRANK after application of residue restraints can be found in Table S4. As shown in Figure S3, the use of residue restraints reduced the total number of docking poses, but the ZRANK score of poses predicted with both docking approaches exhibit similar correlations with DockQ score. The scatterplot of the chosen complex with ideal best pose, AvrPikC-PikhHMA (PDB ID 7a8xEF), illustrates the fact that out of a total of 2000 docking poses re-ranked with ZRANK, 173 satisfied the residue restraints and were thus retained, including the docking pose with the best ZRANK score and a DockQ score of 0.904 (near-native). It is also clear that docking poses with low (favourable) ZRANK score but poor DockQ score (<0.5) were largely excluded by the use of residue restraints. In the case of the scatterplot of the chosen complex with non-ideal best pose, AvrPia-PikpHMA (PDB ID 6q76AB), 298 docking poses satisfied the residue restraints, including the docking pose with the best ZRANK score and a DockQ score of 0.24 (non-near-native). In comparison with blind docking, docking with residue restraints led to improved ranking of near-native docking poses because of the exclusion of poses that do not meet these restraints, resulting in an improvement of pose rankings. For example, in the case of effector-host receptor binding partner complex AvrPikE-PikmHMA (PDB ID 6fubAB), docking pose ranked 14th based on ZRANK score was ranked fourth after the application of residue restraints due to the exclusion of poses ranked at positions 4 to 13 (Table S4). Similar observations were made within the top-15 poses for all bound complexes, except for AvrPikF-OsHIPP19 (PDB ID 7b1iBC), showing that all of the top 15 docking poses based on ZRANK score satisfied the residue restraints. In summary, application of residue restraints excluded unwanted/non-ideal top poses (i.e. high ZRANK score, low DockQ score) and, consequently, improved the ranking of near native poses.

Figure S3. Correlation between ZRANK and DockQ scores for the top 2000 predicted docking poses using ZDOCK score with (in yellow) and without (in grey) the use of residue restraints. The plots shown correspond to effector-host receptor binding partner complexes with ideal best pose, A) AvrPikC-PikhHMA (PDB ID 7a8xEF, and non-ideal best pose, B) AvrPia-PikpHMA (PDB ID 6q76AB). The docking pose with the best ZRANK score is shown in green in each plot. 2000 ZRANK (2k ZRANK) corresponds to the top 2,000 ZDOCK poses re-ranked using ZRANK.

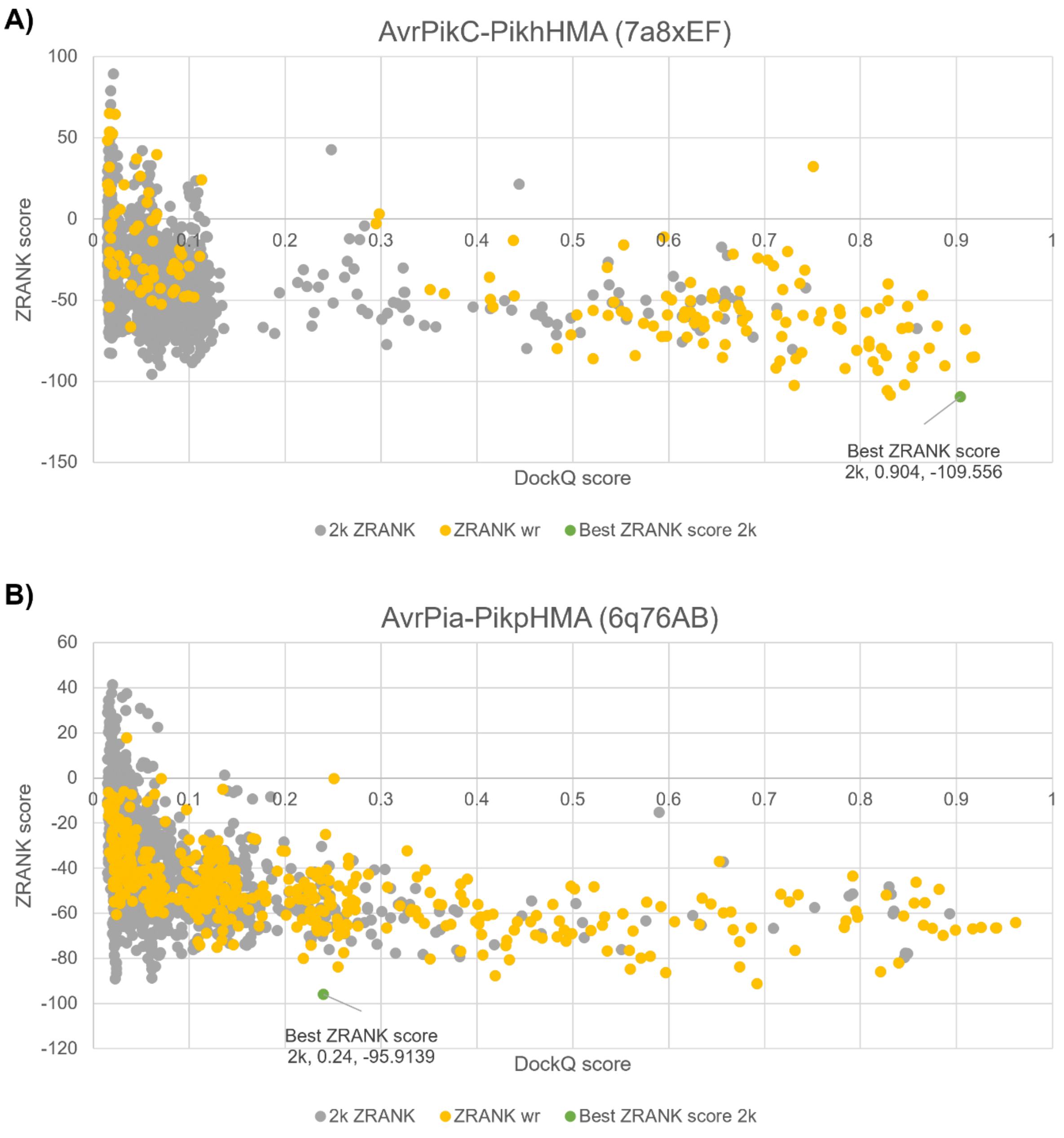

Figure S4. Correlation of ZDOCK and ZRANK scores against DockQ scores for ZDOCK 54,000, ZDOCK 2,000, ZRANK 2,000 docking poses. The best ZDOCK and ZRANK docking poses (in coloured dots) are shown as indicated by the figure legend. ZRANK 2,000 refers to the top 2,000 ZDOCK poses re-ranked using ZRANK.

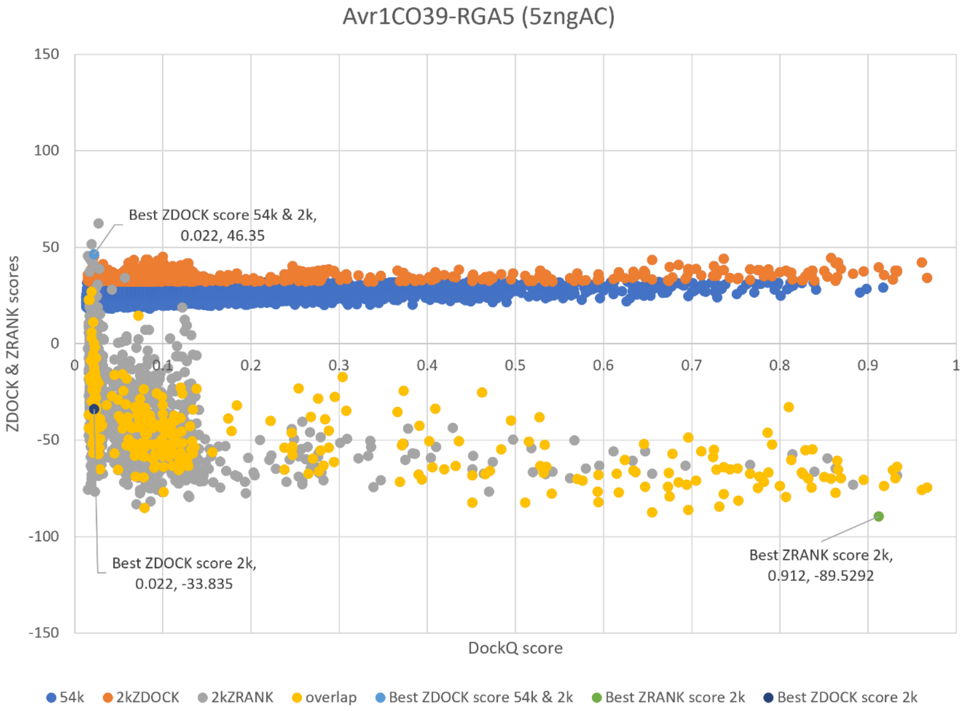

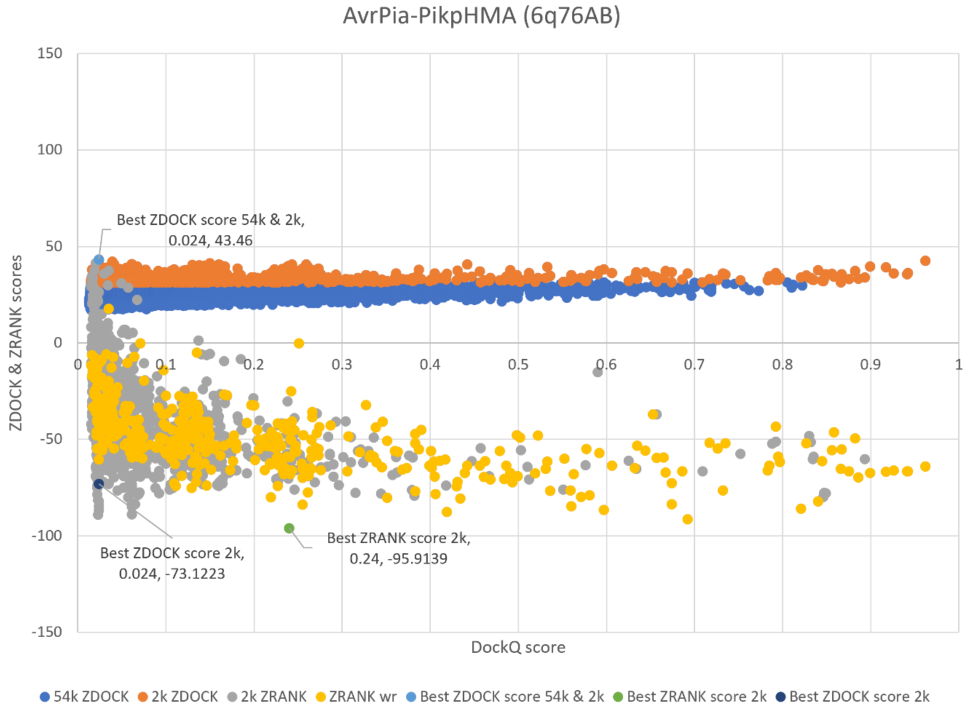

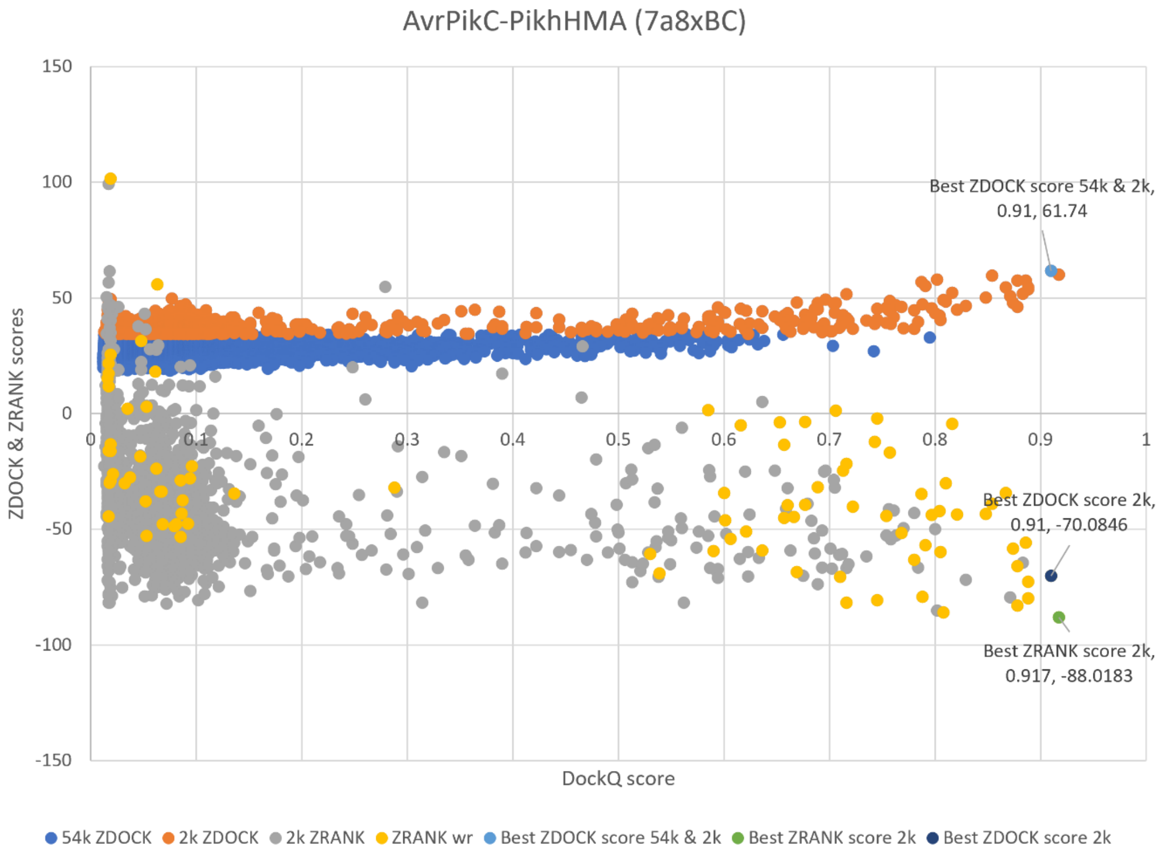
AvrPikC-PikhHMA (7a8xBC)
150
100
50
0
-50
-100
-150
0
0.1
0.2
0.3
0.4
0.5
0.6
0.7
0.8
0.9
1
ZDOCK & ZRANK scores
DockQ score
Best ZDOCK score 54k & 2k, 0.91, 61.74
Best ZDOCK score 2k, 0.91, -70.0846
Best ZRANK score 2k, 0.917, -88.0183
54k ZDOCK
2k ZDOCK
2k ZRANK
ZRANK wr
Best ZDOCK score 54k & 2k
Best ZRANK score 2k
Best ZDOCK score 2k

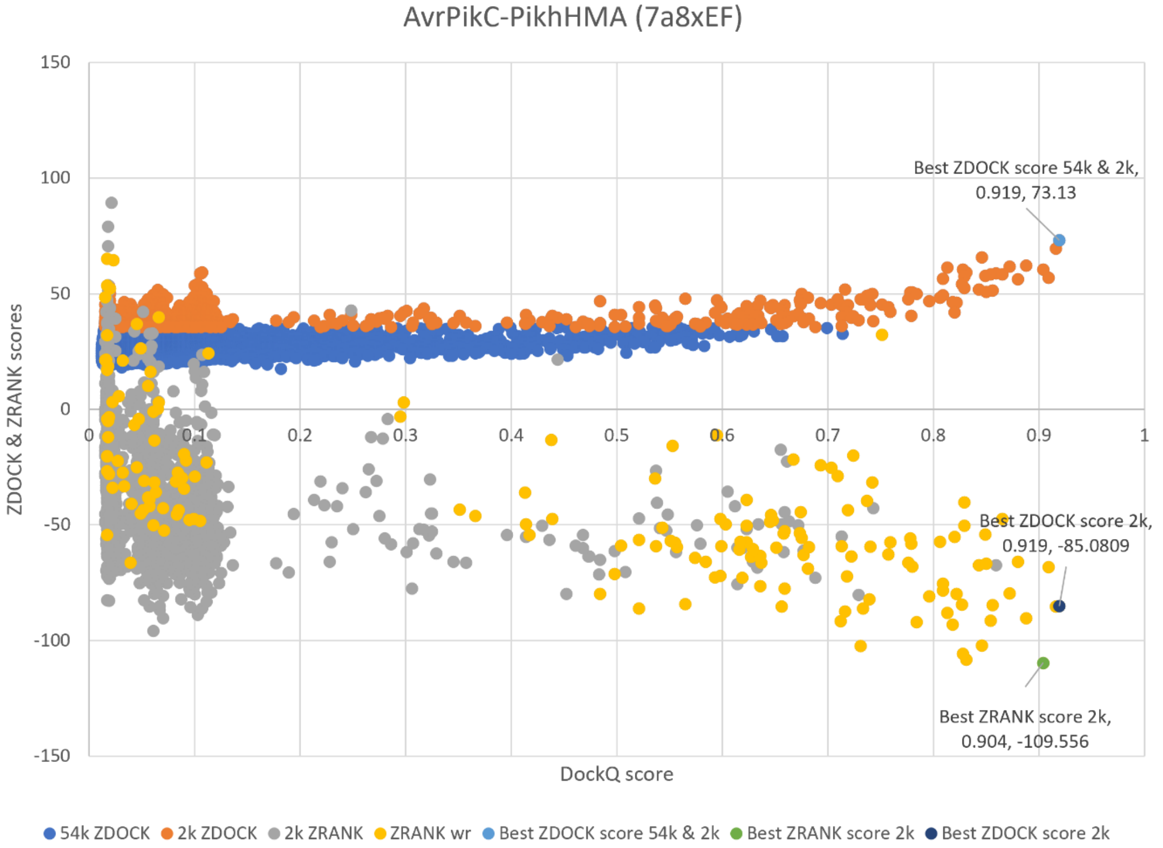
AvrPikC-PikhHMA (7a8xEF)
150
100
50
0
-50
-100
-150
0
0.1
0.2
0.3
0.4
0.5
0.6
0.7
0.8
0.9
1
ZDOCK & ZRANK scores
DockQ score
Best ZDOCK score 54k & 2k, 0.919, 73.13
Best ZDOCK score 2k, 0.919, -85.0809
Best ZRANK score 2k, 0.904, -109.556
54k ZDOCK
2k ZDOCK
2k ZRANK
ZRANK wr
Best ZDOCK score 54k & 2k
Best ZRANK score 2k
Best ZDOCK score 2k

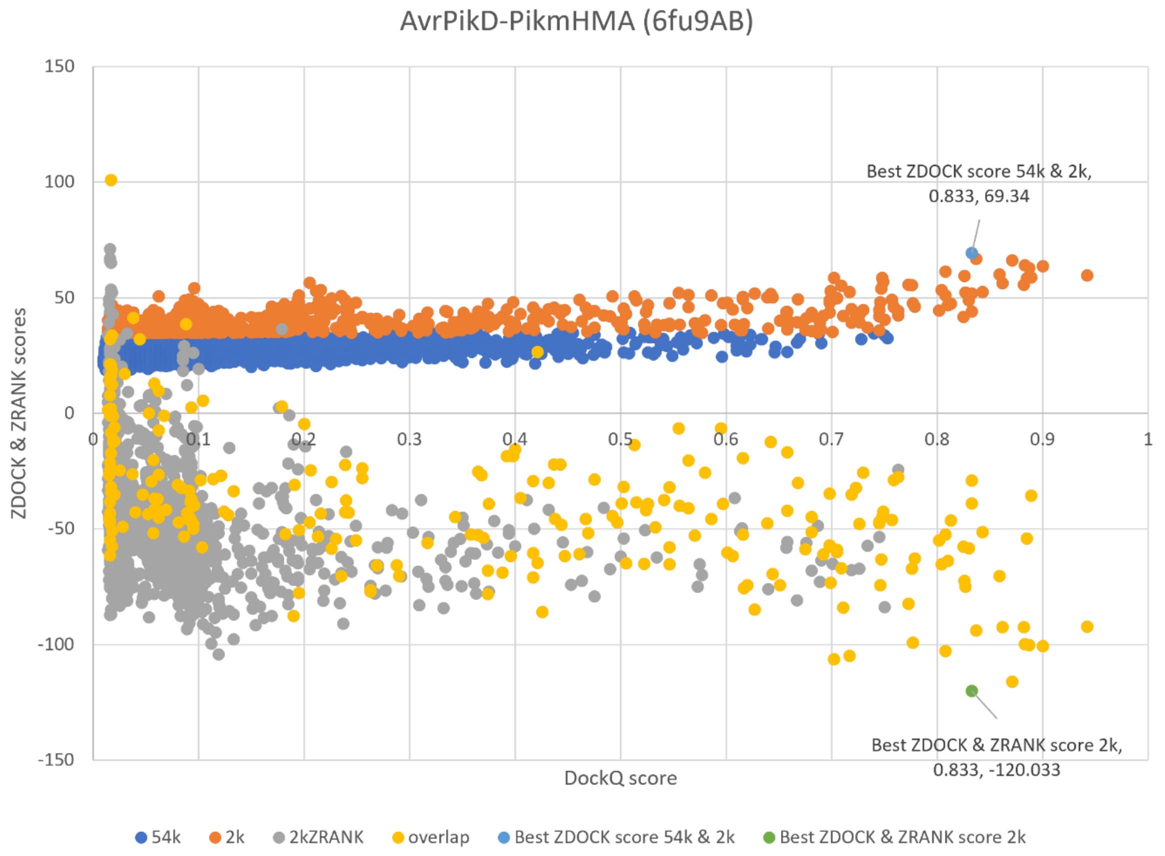
AvrPikD-PikmHMA (6fu9AB)
ZDOCK & ZRANK scores
DockQ score
Best ZDOCK score 54k & 2k, 0.833, 69.34
Best ZDOCK & ZRANK score 2k, 0.833, -120.033
54k
2k
2kZRANK
overlap
Best ZDOCK score 54k & 2k
Best ZDOCK & ZRANK score 2k

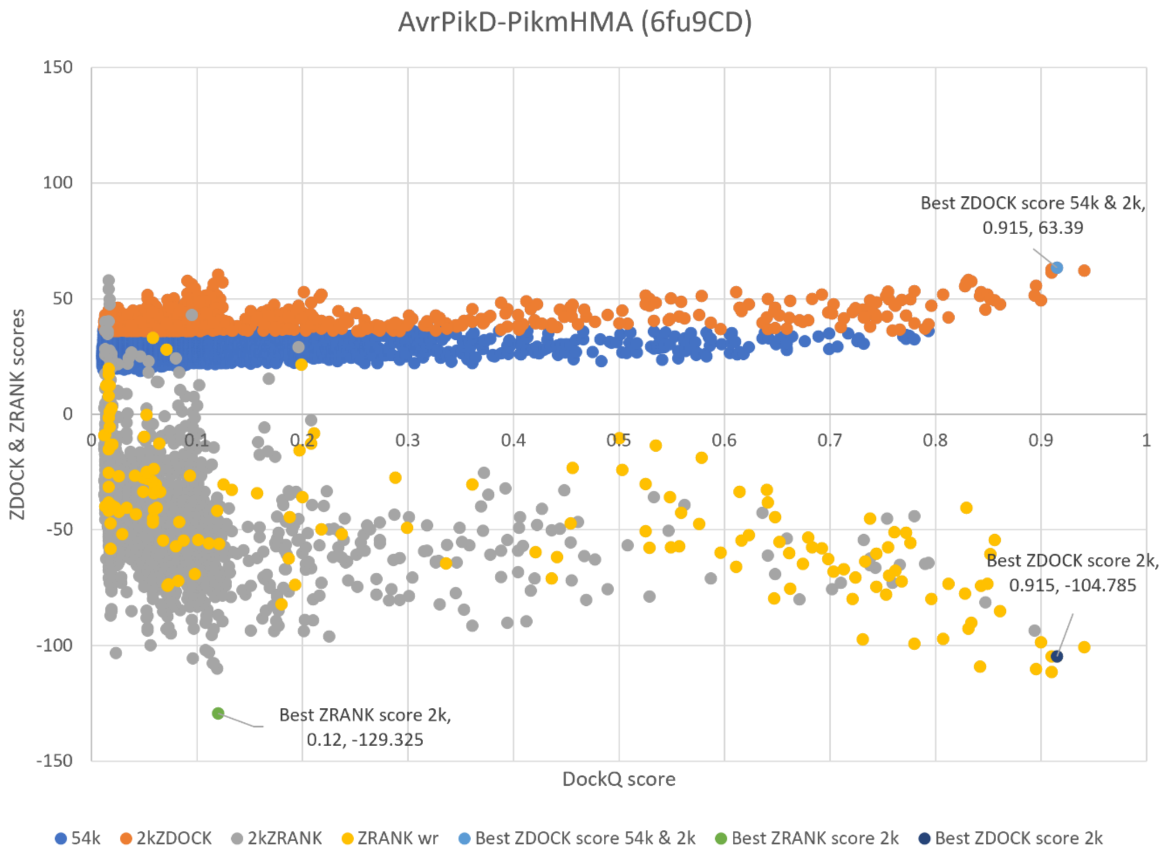
AvrPikD-PikmHMA (6fu9CD)
ZDOCK & ZRANK scores
DockQ score
Best ZDOCK score 54k & 2k, 0.915, 63.39
Best ZDOCK score 2k, 0.915, -104.785
Best ZRANK score 2k, 0.12, -129.325
54k
2kZDOCK
2kZRANK
ZRANK wr
Best ZDOCK score 54k & 2k
Best ZRANK score 2k
Best ZDOCK score 2k

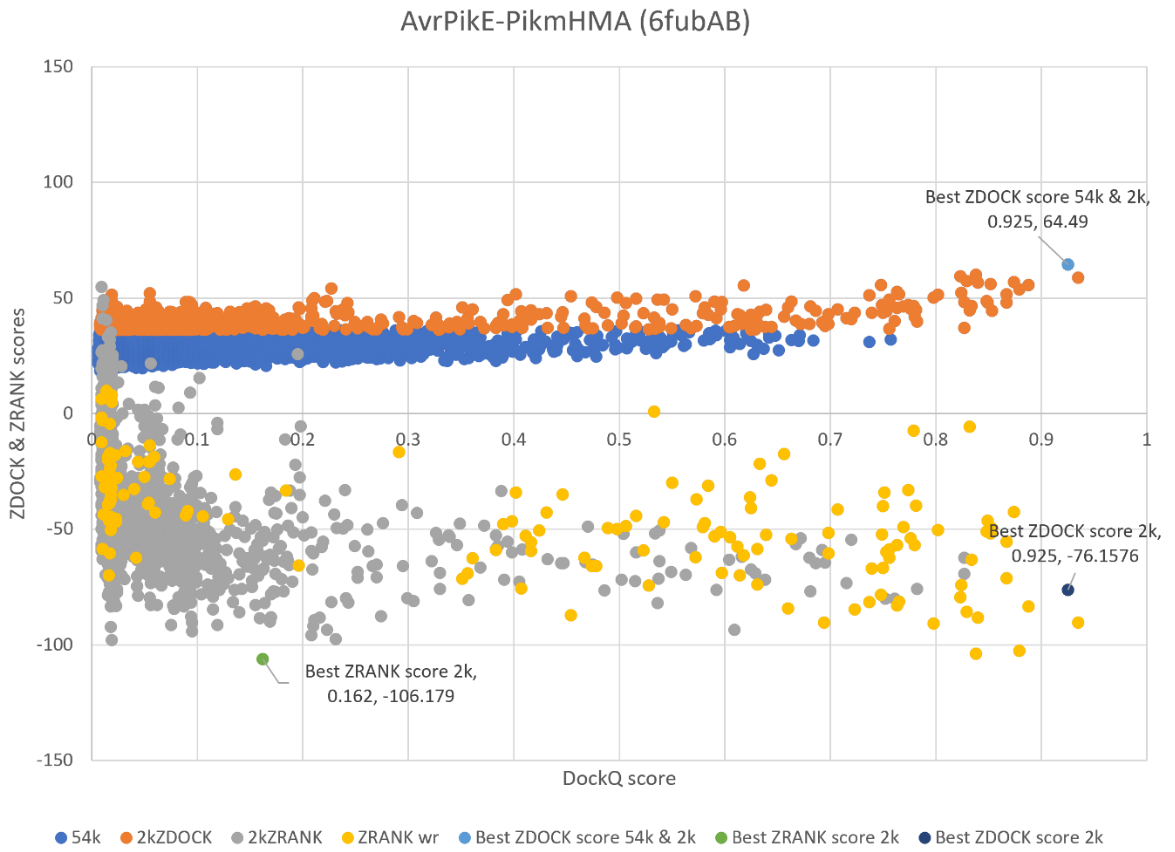
AvrPikE-PikmHMA (6fubAB)
ZDOCK & ZRANK scores
DockQ score
Best ZDOCK score 54k & 2k,
0.925, 64.49
Best ZDOCK score 2k,
0.925, -76.1576
Best ZRANK score 2k,
0.162, -106.179
54k
2kZDOCK
2kZRANK
ZRANK wr
Best ZDOCK score 54k & 2k
Best ZRANK score 2k
Best ZDOCK score 2k

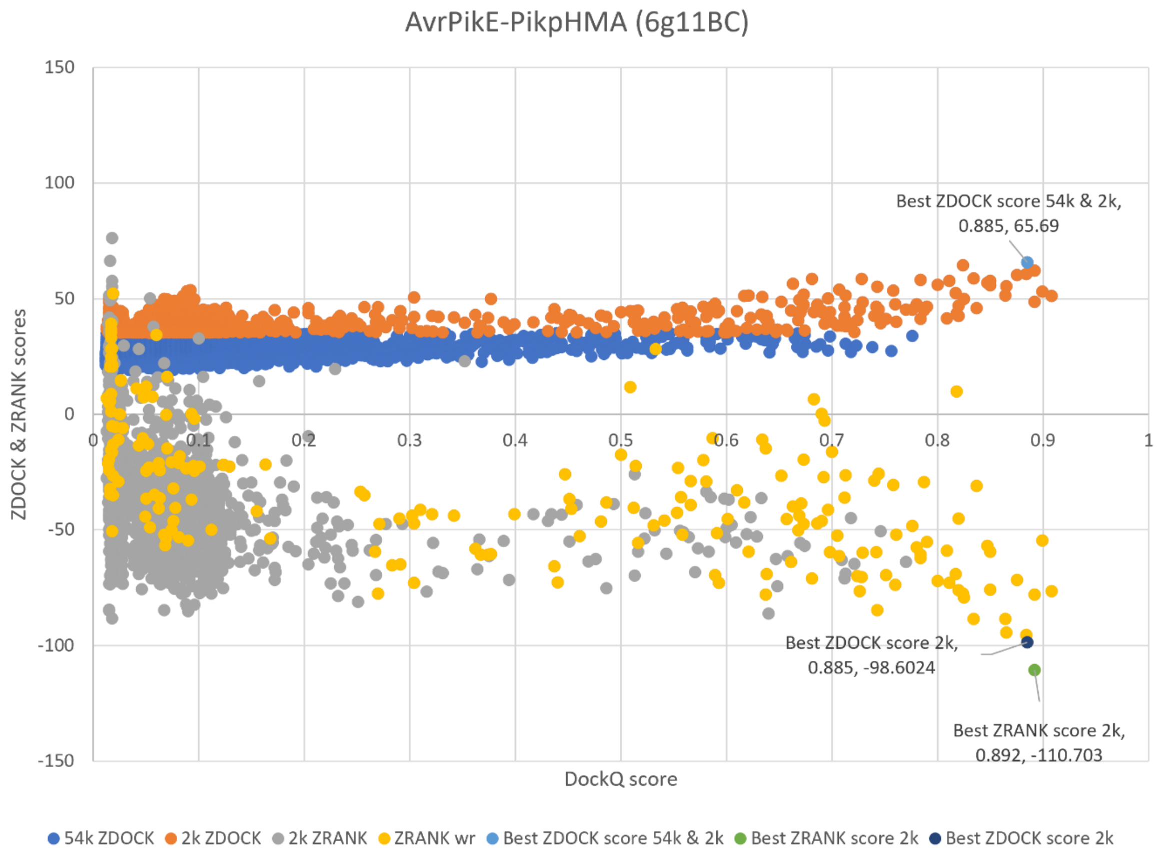
AvrPikE-PikpHMA (6g11BC)
ZDOCK & ZRANK scores
DockQ score
Best ZDOCK score 54k & 2k,
0.885, 65.69
Best ZDOCK score 2k,
0.885, -98.6024
Best ZRANK score 2k,
0.892, -110.703
54k ZDOCK
2k ZDOCK
2k ZRANK
ZRANK wr
Best ZDOCK score 54k & 2k
Best ZRANK score 2k
Best ZDOCK score 2k

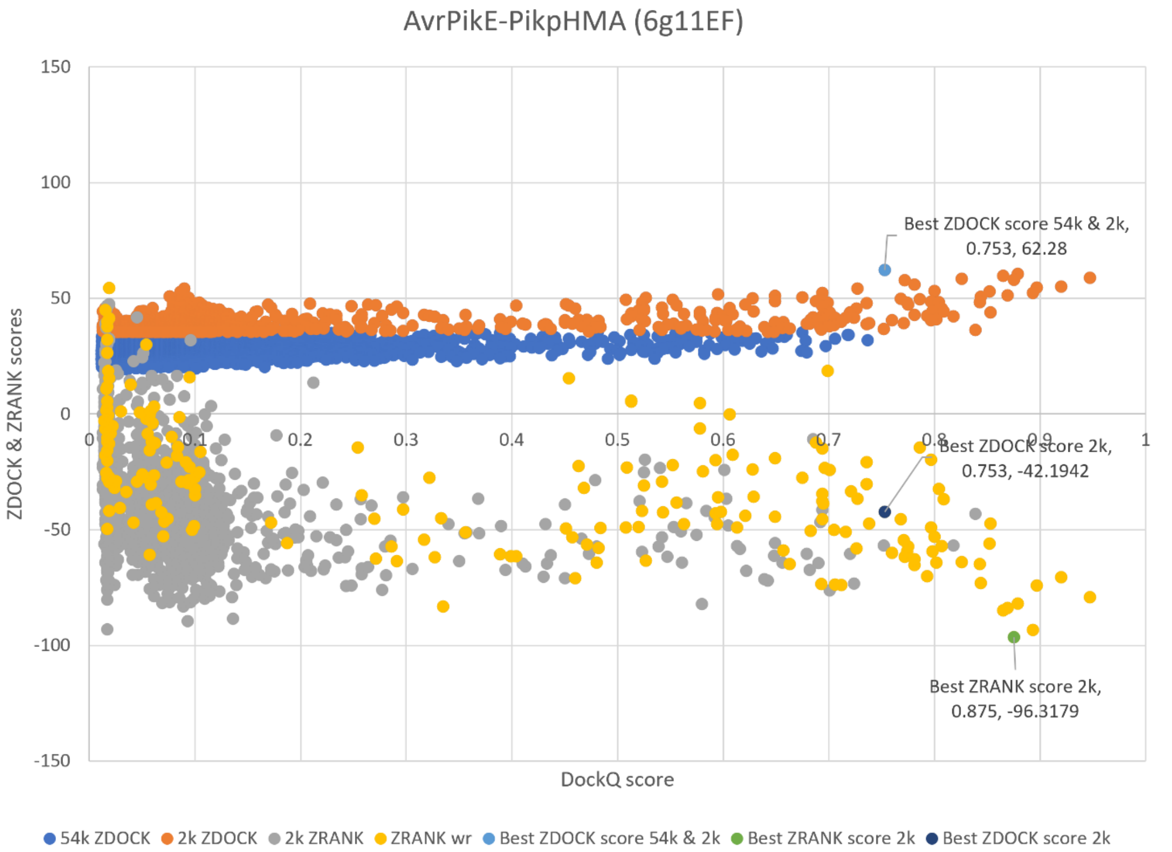
AvrPikE-PikpHMA (6g11EF)
150
100
50
0
-50
-100
-150
0
0.1
0.2
0.3
0.4
0.5
0.6
0.7
0.8
0.9
1
ZDOCK & ZRANK scores
DockQ score
Best ZDOCK score 54k & 2k, 0.753, 62.28
Best ZDOCK score 2k, 0.753, -42.1942
Best ZRANK score 2k, 0.875, -96.3179
54k ZDOCK
2k ZDOCK
2k ZRANK
ZRANK wr
Best ZDOCK score 54k & 2k
Best ZRANK score 2k
Best ZDOCK score 2k

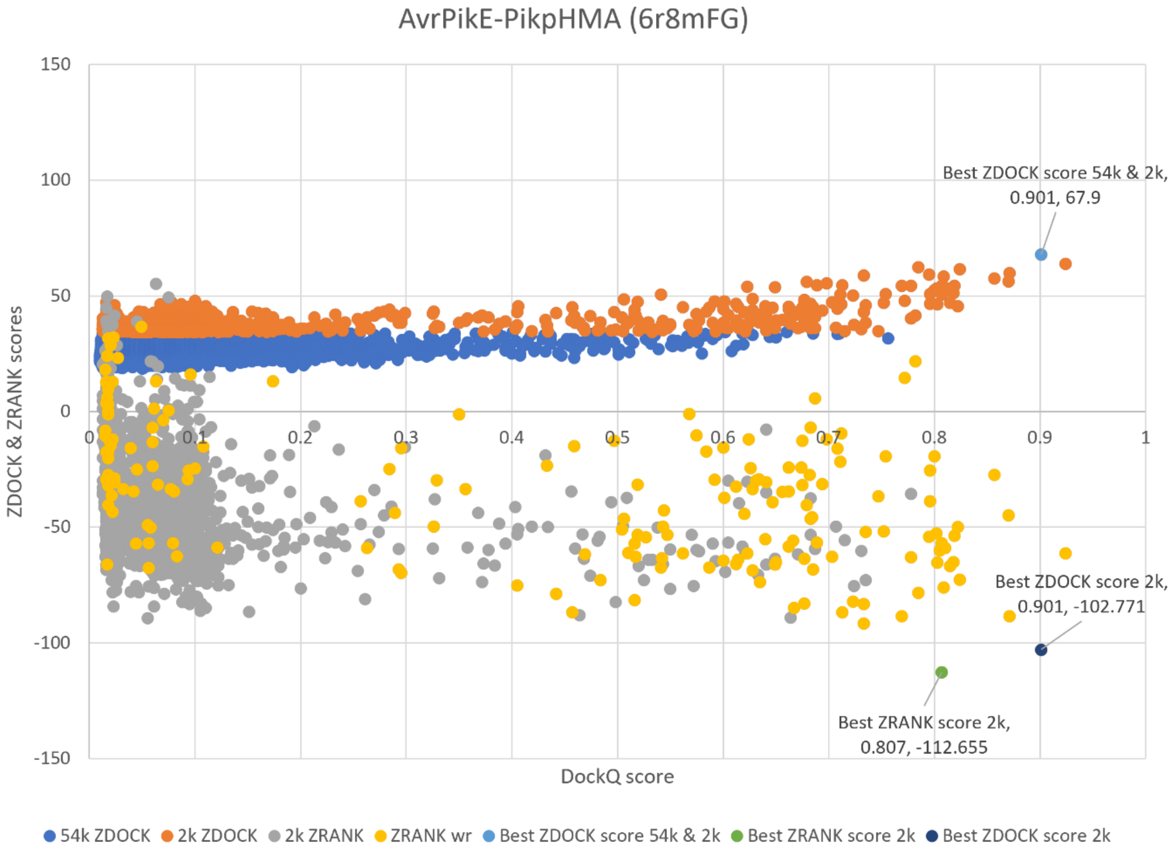
AvrPikE-PikpHMA (6r8mFG)
150
100
50
0
-50
-100
-150
0
0.1
0.2
0.3
0.4
0.5
0.6
0.7
0.8
0.9
1
ZDOCK & ZRANK scores
DockQ score
Best ZDOCK score 54k & 2k, 0.901, 67.9
Best ZDOCK score 2k, 0.901, -102.771
Best ZRANK score 2k, 0.807, -112.655
54k ZDOCK
2k ZDOCK
2k ZRANK
ZRANK wr
Best ZDOCK score 54k & 2k
Best ZRANK score 2k
Best ZDOCK score 2k

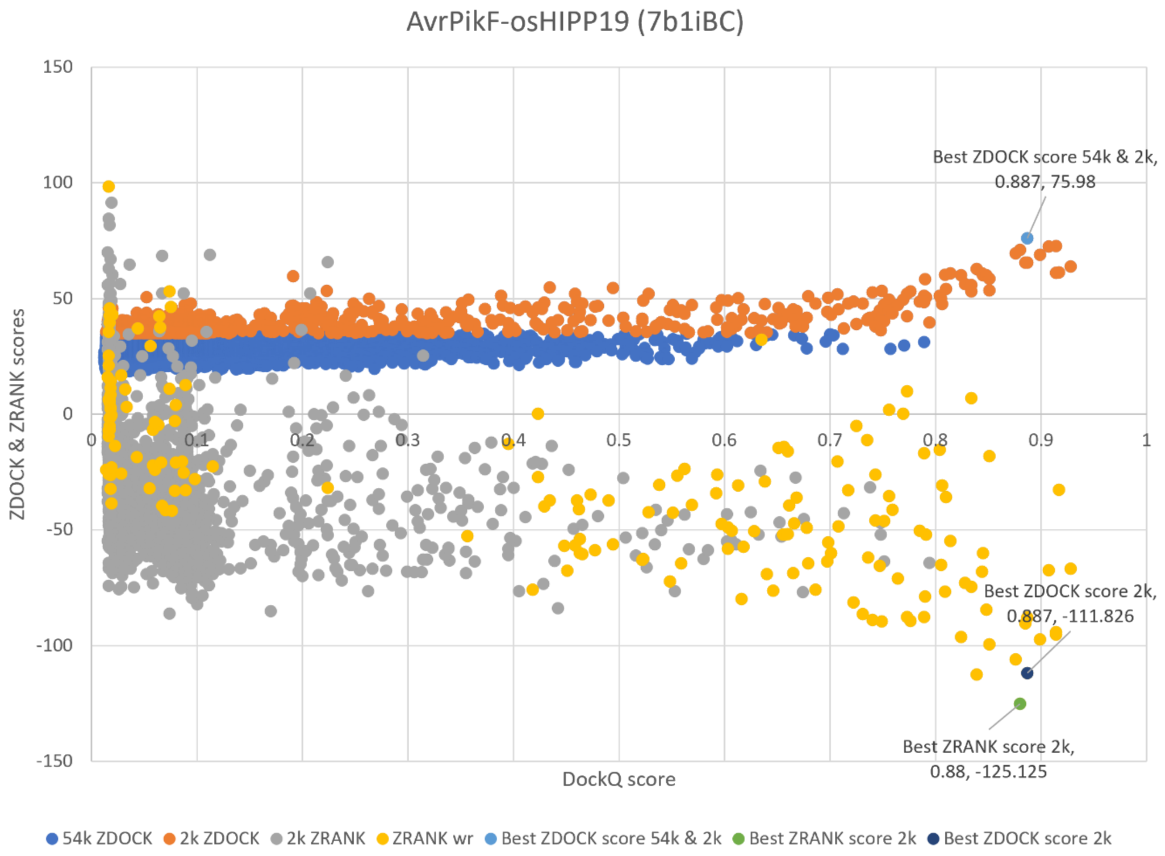
AvrPikF-osHIPP19 (7b1iBC)
ZDOCK & ZRANK scores
DockQ score
Best ZDOCK score 54k & 2k, 0.887, 75.98
Best ZDOCK score 2k, 0.887, -111.826
Best ZRANK score 2k, 0.88, -125.125
54k ZDOCK
2k ZDOCK
2k ZRANK
ZRANK wr
Best ZDOCK score 54k & 2k
Best ZRANK score 2k
Best ZDOCK score 2k

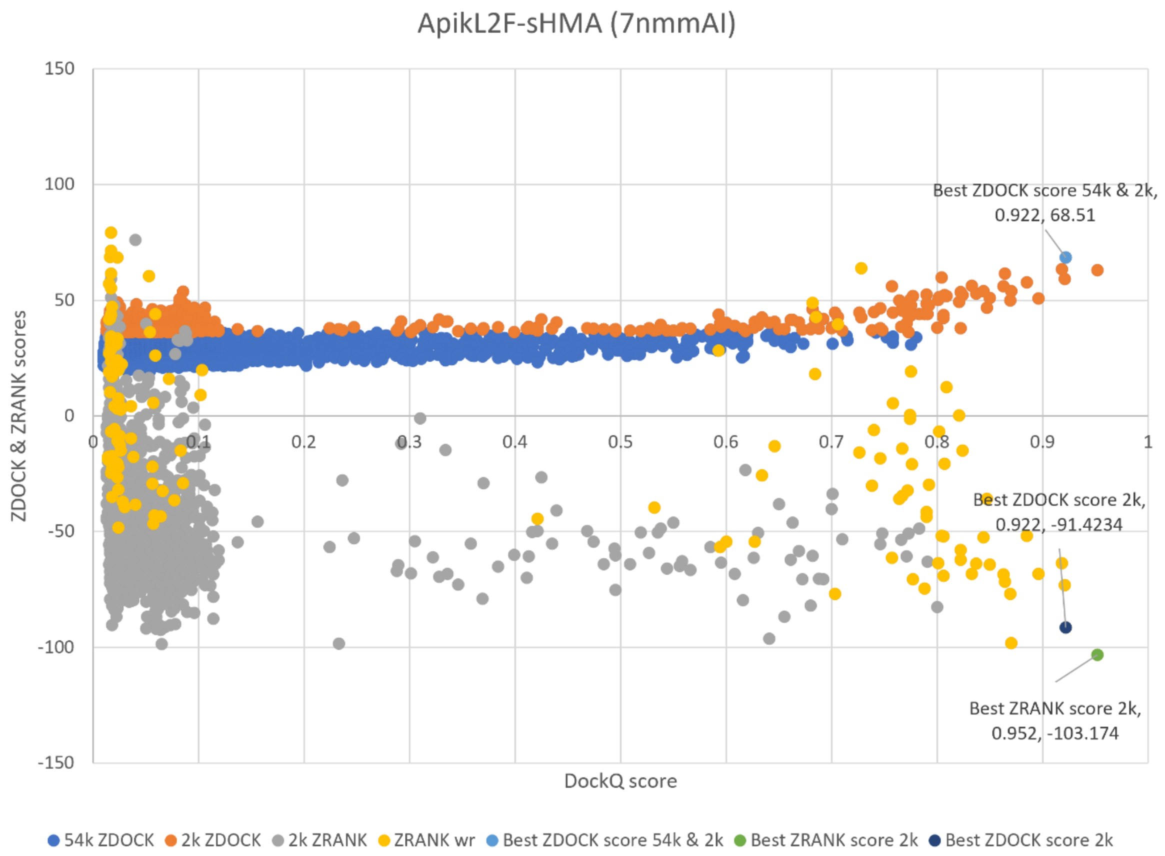
ApikL2F-sHMA (7nmmAI)
ZDOCK & ZRANK scores
DockQ score
Best ZDOCK score 54k & 2k, 0.922, 68.51
Best ZDOCK score 2k, 0.922, -91.4234
Best ZRANK score 2k, 0.952, -103.174
54k ZDOCK
2k ZDOCK
2k ZRANK
ZRANK wr
Best ZDOCK score 54k & 2k
Best ZRANK score 2k
Best ZDOCK score 2k

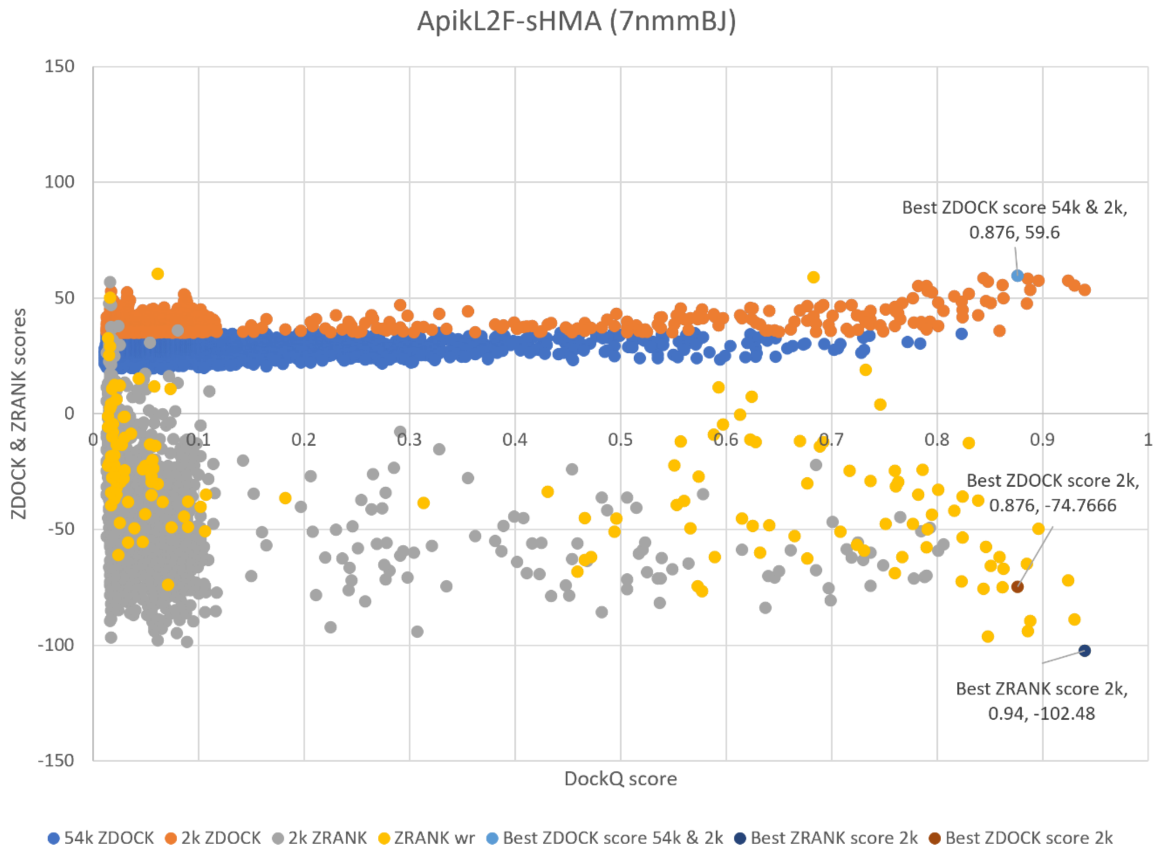
ApikL2F-sHMA (7nmmBJ)
150
100
50
0
-50
-100
-150
0
0.1
0.2
0.3
0.4
0.5
0.6
0.7
0.8
0.9
1
ZDOCK & ZRANK scores
DockQ score
Best ZDOCK score 54k & 2k,
0.876, 59.6
Best ZDOCK score 2k,
0.876, -74.7666
Best ZRANK score 2k,
0.94, -102.48
54k ZDOCK
2k ZDOCK
2k ZRANK
ZRANK wr
Best ZDOCK score 54k & 2k
Best ZRANK score 2k
Best ZDOCK score 2k

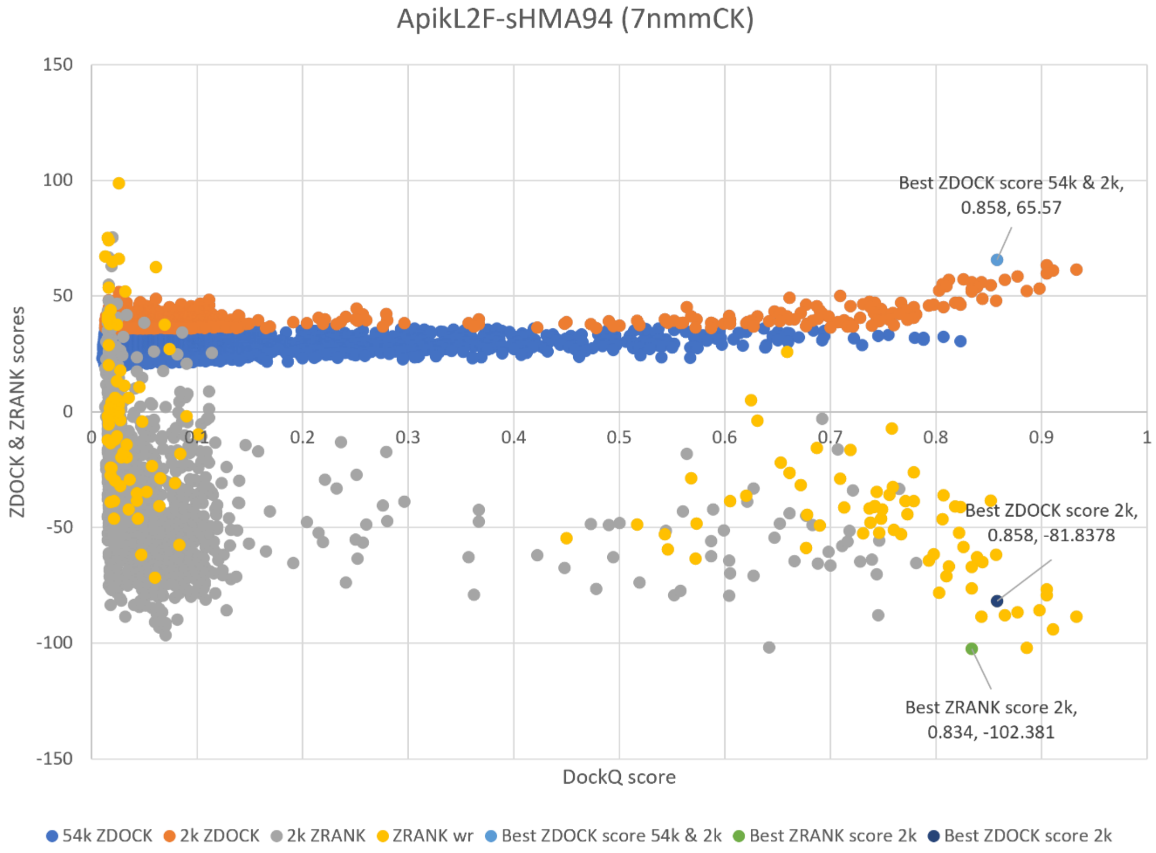
ApikL2F-sHMA94 (7nmmCK)
150
100
50
0
-50
-100
-150
0
0.1
0.2
0.3
0.4
0.5
0.6
0.7
0.8
0.9
1
ZDOCK & ZRANK scores
DockQ score
Best ZDOCK score 54k & 2k,
0.858, 65.57
Best ZDOCK score 2k,
0.858, -81.8378
Best ZRANK score 2k,
0.834, -102.381
54k ZDOCK
2k ZDOCK
2k ZRANK
ZRANK wr
Best ZDOCK score 54k & 2k
Best ZRANK score 2k
Best ZDOCK score 2k

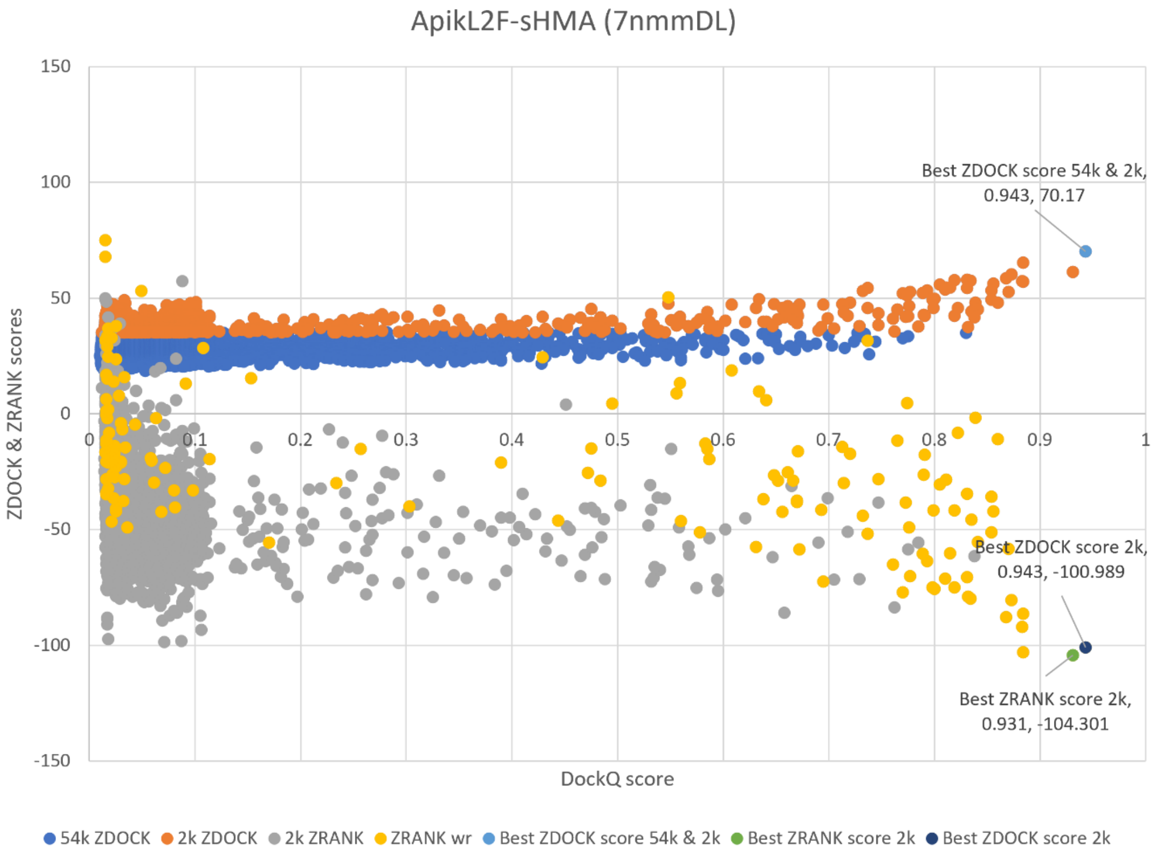
ApikL2F-sHMA (7nmmDL)
ZDOCK & ZRANK scores
DockQ score
Best ZDOCK score 54k & 2k, 0.943, 70.17
Best ZDOCK score 2k, 0.943, -100.989
Best ZRANK score 2k, 0.931, -104.301
54k ZDOCK
2k ZDOCK
2k ZRANK
ZRANK wr
Best ZDOCK score 54k & 2k
Best ZRANK score 2k
Best ZDOCK score 2k

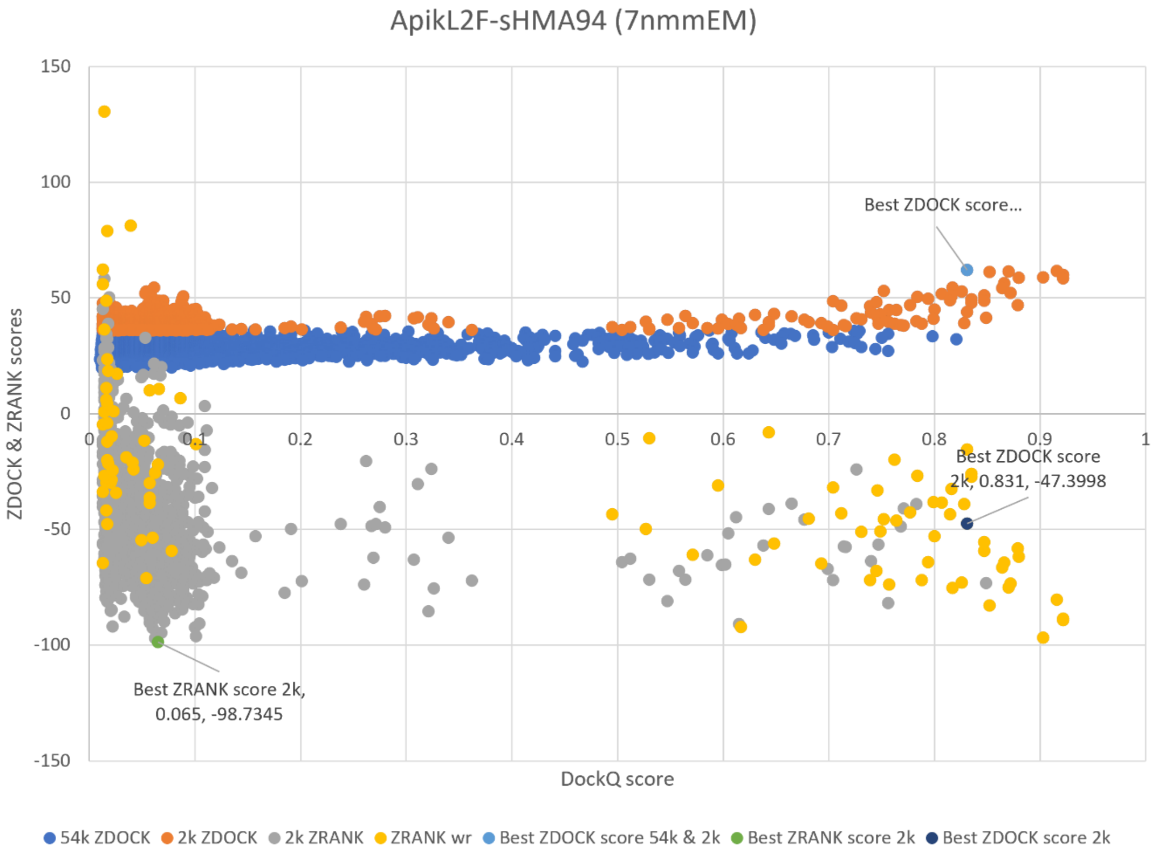
ApikL2F-sHMA94 (7nmmEM)
ZDOCK & ZRANK scores
DockQ score
Best ZDOCK score...
Best ZDOCK score 2k, 0.831, -47.3998
Best ZRANK score 2k, 0.065, -98.7345
54k ZDOCK
2k ZDOCK
2k ZRANK
ZRANK wr
Best ZDOCK score 54k & 2k
Best ZRANK score 2k
Best ZDOCK score 2k

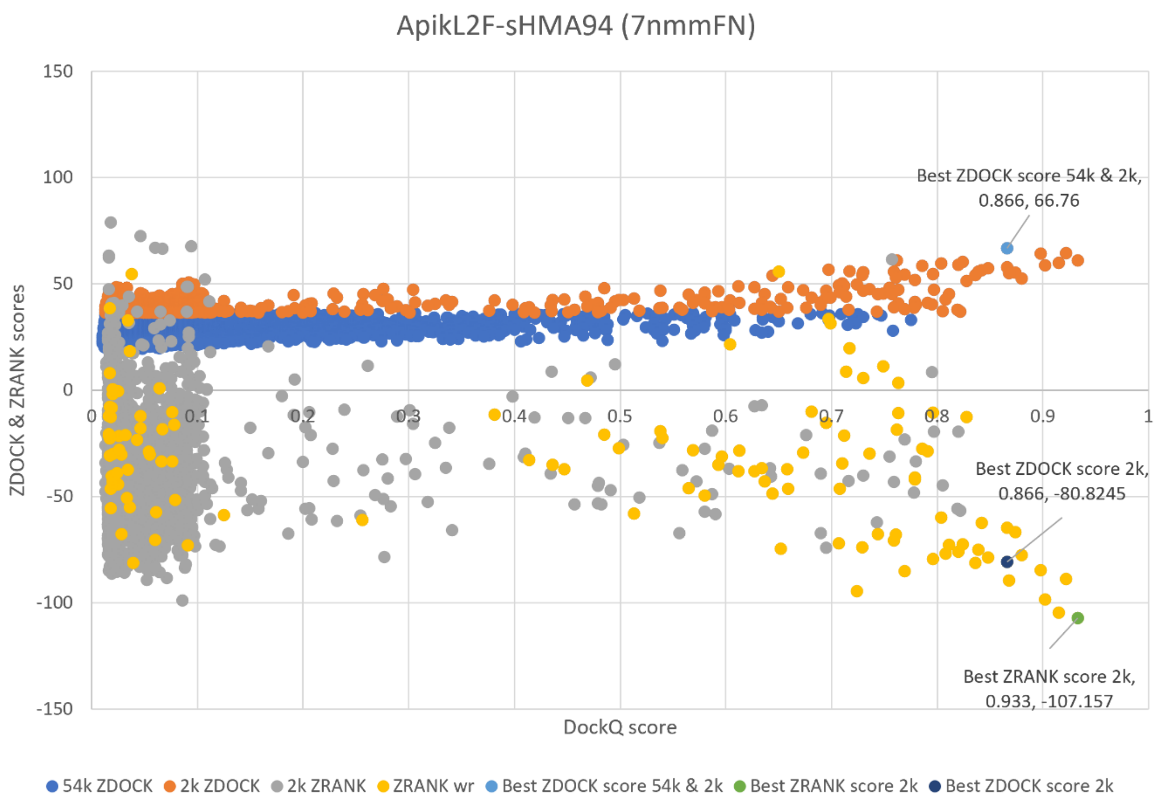
ApikL2F-sHMA94 (7nmmFN)
150
100
50
0
-50
-100
-150
0
0.1
0.2
0.3
0.4
0.5
0.6
0.7
0.8
0.9
1
ZDOCK & ZRANK scores
DockQ score
Best ZDOCK score 54k & 2k, 0.866, 66.76
Best ZDOCK score 2k, 0.866, -80.8245
Best ZRANK score 2k, 0.933, -107.157
54k ZDOCK
2k ZDOCK
2k ZRANK
ZRANK wr
Best ZDOCK score 54k & 2k
Best ZRANK score 2k
Best ZDOCK score 2k

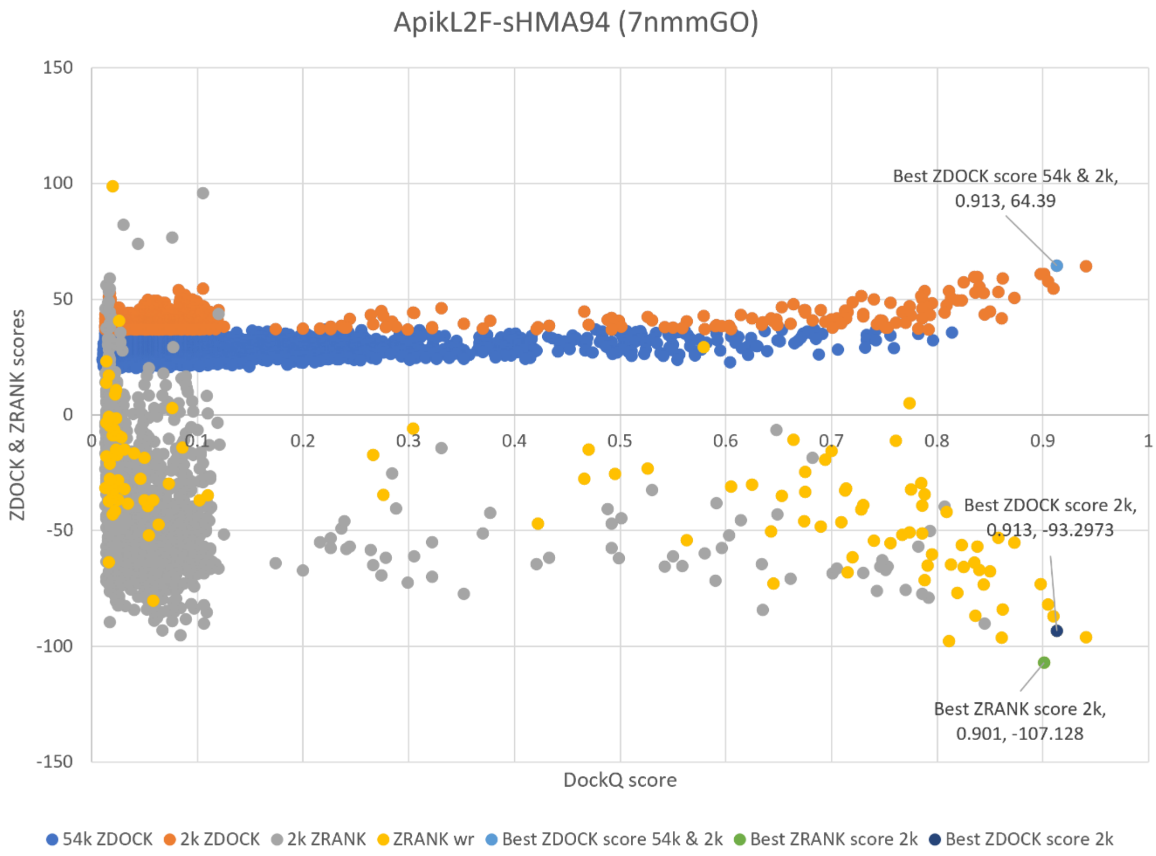
ApikL2F-sHMA94 (7nmmGO)
150
100
50
0
-50
-100
-150
0
0.1
0.2
0.3
0.4
0.5
0.6
0.7
0.8
0.9
1
ZDOCK & ZRANK scores
DockQ score
Best ZDOCK score 54k & 2k, 0.913, 64.39
Best ZDOCK score 2k, 0.913, -93.2973
Best ZRANK score 2k, 0.901, -107.128
54k ZDOCK
2k ZDOCK
2k ZRANK
ZRANK wr
Best ZDOCK score 54k & 2k
Best ZRANK score 2k
Best ZDOCK score 2k

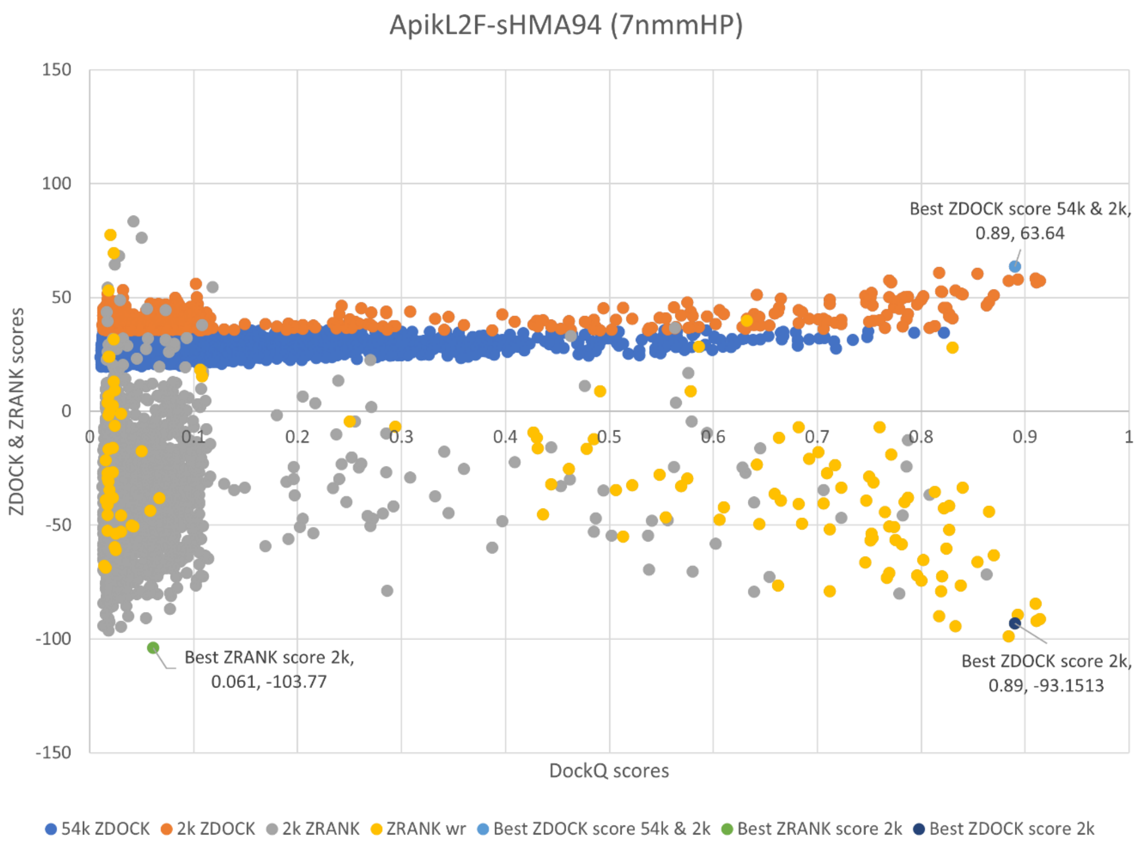
ApikL2F-sHMA94 (7nmmHP)
150
100
50
0
-50
-100
-150
0
0.1
0.2
0.3
0.4
0.5
0.6
0.7
0.8
0.9
1
ZDOCK & ZRANK scores
DockQ scores
Best ZDOCK score 54k & 2k, 0.89, 63.64
Best ZRANK score 2k, 0.061, -103.77
Best ZDOCK score 2k, 0.89, -93.1513
54k ZDOCK
2k ZDOCK
2k ZRANK
ZRANK wr
Best ZDOCK score 54k & 2k
Best ZRANK score 2k
Best ZDOCK score 2k

Figure S5. Correlation between ELE CCharPPI scoring function score and DockQ score for all complexes: AvrPikC-PikhHMA (PDB ID 7a8xBC) in blue square, AvrPikD-PikmHMA (PDB ID 6fu9AB) in orange square, AvrPikE-PikmHMA (PDB ID 6g11BC) in green triangle, AvrPikE-PikmHMA (PDB ID 6g11EF) in grey triangle, AvrPikE-PikmHMA (PDB ID 6r8mFG) in blue circle, AvrPikF-OsHIPP19 (PDB ID 7b1iBC) in yellow cross, ApikL2F-sHMA94 (PDB ID 7nmmAI) in blue asterisk, ApikL2F-sHMA94 (PDB ID 7nmmCK) in green circle, ApikL2F-sHMA94 (PDB ID 7nmmDL) in blue plus symbol, ApikL2F-sHMA94 (PDB ID 7nmmFN) in orange short dash, ApikL2F-sHMA94 (PDB ID 7nmmGO) in grey long dash, ApikL2F-sHMA94 (PDB ID 7nmmHP) in yellow diamond.

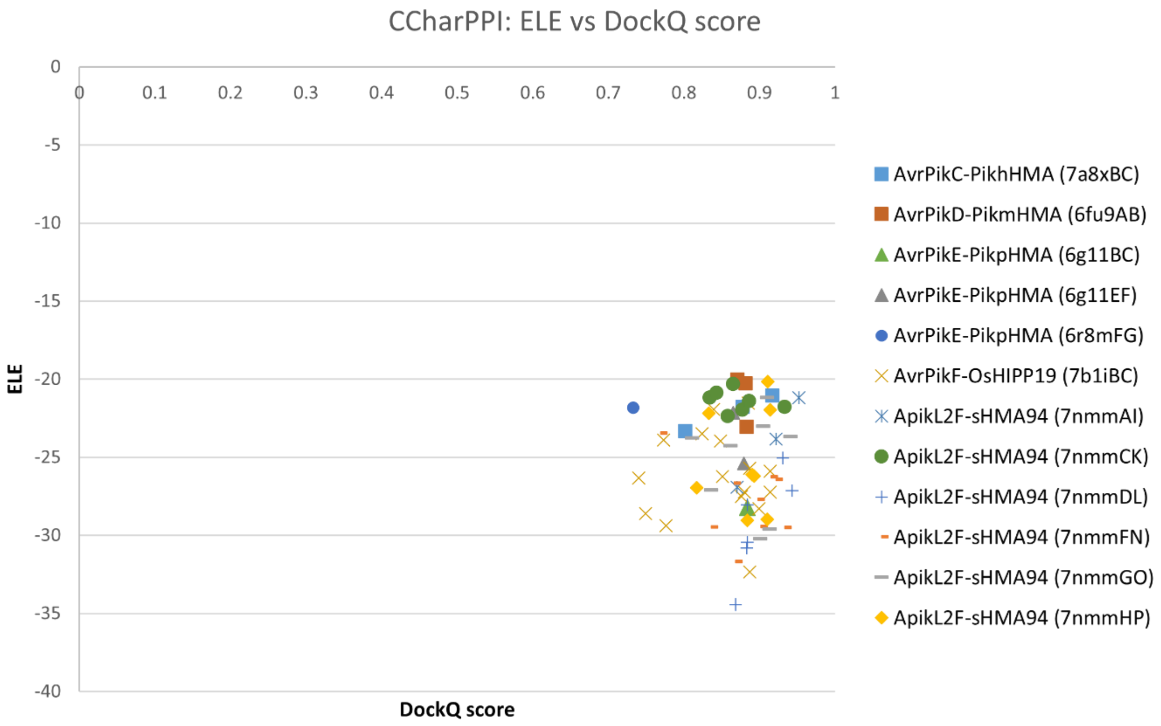

Supplement: Supplementary file 1 [file ijms-24-15239-s001.zip › ijms-2670759-supplementary.pdf]
